# Genome Comparison of *Candida orthopsilosis* Clinical Strains Reveals the Existence of Hybrids between Two Distinct Subspecies

**DOI:** 10.1093/gbe/evu082

**Published:** 2014-04-18

**Authors:** Leszek P. Pryszcz, Tibor Németh, Attila Gácser, Toni Gabaldón

**Affiliations:** ^1^Bioinformatics and Genomics Programme, Centre for Genomic Regulation (CRG), Barcelona, Spain; ^2^Universitat Pompeu Fabra (UPF), Barcelona, Spain; ^3^Department of Microbiology, University of Szeged, Hungary; ^4^Institució Catalana de Recerca i Estudis Avançats (ICREA), Barcelona, Spain

**Keywords:** *Candida orthopsilosis*, hybridization, pathogens, genome sequencing, fungi

## Abstract

The *Candida parapsilosis* species complex comprises a group of emerging human pathogens of varying virulence. This complex was recently subdivided into three different species: *C. parapsilosis* sensu stricto, *C. metapsilosis*, and *C. orthopsilosis*. Within the latter, at least two clearly distinct subspecies seem to be present among clinical isolates (Type 1 and Type 2). To gain insight into the genomic differences between these subspecies, we undertook the sequencing of a clinical isolate classified as Type 1 and compared it with the available sequence of a Type 2 clinical strain. Unexpectedly, the analysis of the newly sequenced strain revealed a highly heterozygous genome, which we show to be the consequence of a hybridization event between both identified subspecies. This implicitly suggests that *C. orthopsilosis* is able to mate, a so-far unanswered question. The resulting hybrid shows a chimeric genome that maintains a similar gene dosage from both parental lineages and displays ongoing loss of heterozygosity. Several of the differences found between the gene content in both strains relate to virulent-related families, with the hybrid strain presenting a higher copy number of genes coding for efflux pumps or secreted lipases. Remarkably, two clinical strains isolated from distant geographical locations (Texas and Singapore) are descendants of the same hybrid line, raising the intriguing possibility of a relationship between the hybridization event and the global spread of a virulent clone.

## Introduction

Although *Candida albicans* remains the main cause of invasive candidiasis cases, clinical data indicate that the members of the *C. parapsilosis* complex are significant pathogens with increasing prevalence (van Asbeck et al. 2009). This species complex was recently recognized to contain three closely related species, formerly distinguished as *C. parapsilosis* group I, group II, and group III, which became known as *C. parapsilosis* sensu stricto, *C. orthopsilosis*, and *C. metapsilois*, respectively (Tavanti et al. 2005). Although the three species are closely related, they differ markedly in their clinical prevalence, virulence, and antifungal susceptibility. However, all three species are able to cause serious conditions with various clinical manifestations, including fungaemia (Lockhart et al. 2008). Several epidemiological studies indicate that the majority of *C. parapsilosis* complex infections are caused by *C. parapsilosis* sensu stricto isolates, and *C. orthopsilosis* is responsible for about 1–10% of cases depending on the geographic region (Lockhart et al. 2008). However, *C. orthopsilosis* is more frequently identified than *C. metapsilosis* (Bonfietti et al. 2012; Riccombeni et al. 2012), although both species have been associated with several outbreaks of infection in addition to sporadic cases (Lockhart et al. 2008; Ge et al. 2012; Taj-Aldeen et al. 2013). Although the commensal status of *C. parapsilosis* sensu stricto in humans is well known, that of the other two species from the *C. parapsilosis* complex has not been confirmed yet, although a metagenomic study indicated the presence of *C. metapsilosis* in the oral cavity of healthy human subjects (Ghannoum et al. 2010).

Despite their clinical importance, there are relatively few studies investigating the virulence of the *C. parapsilosis* sensu lato species, especially for *C*. *orthopsilosis* and *C*. *metapsilosis*. To date, only few studies have been conducted to compare the virulence of *C*. *parapsilosis* sensu stricto, *C. orthopsilosis*, and *C. metapsilosis* isolates. Most studies generally indicate that, for the conditions tested, *C. orthopsilosis* displays virulence and adhesion properties similar to *C. parapsilosis*, whereas *C. metapsilosis* is the least virulent species (Gácser, Schäfer, et al. 2007; Butler et al. 2009; Orsi et al. 2010; Bertini et al. 2013). The most extensive study to date examining the virulence of the three species using in vitro and in vivo infection systems determined that *C. orthopsilosis* and *C. metapsilosis* isolates were less resistant to killing by human macrophages compared with *C. parapsilosis* sensu stricto strains (Németh et al. 2013). In addition, this study analyzed the producing capacity, in several strains of each species, of extracellular protease and lipase (both known as important virulence factors during *Candida* infections [Monod and Borg-von 2002; Gácser, Trofa, et al. 2007]). Their results showed that, although the majority of *C. parapsilosis* sensu stricto strains were positive for both lipase and protease production, only a small number of *C. orthopsilosis* and none of the *C. metapsilosis* isolates were able to produce lipases.

Considering that virulence properties can vary significantly among strains of the same species, it is important to study the detailed genetic background of pathogenic isolates. This aspect seems to be of particular importance for *C. orthopsilosis*, where initial analyses of ITS region and MAT-cassette genes have shown that this species is formed by at least two clearly distinguishable subspecies: Called Type 1 and Type 2 (Sai et al. 2011). The genome sequence of *C. parapsilosis* sensu stricto has been available since 2009 (Butler et al. 2009), facilitating numerous studies on the biology and virulence of this species. Recently, the first genome-wide comparison of several *C. parapsilosis* strains was published, shedding light onto the population structure of this species (Pryszcz et al. 2013). The genome of a Type 2 *C. orthopsilosis* was sequenced only in 2012 (Riccombeni et al. 2012), which served to compare it extensively with that of *C. parapsilosis*. The main differences found included an expansion of the Hyr/Iff family of cell wall genes and the JEN family of monocarboxylic transporters in *C. parapsilosis* relative to *C. orthopsilosis*. However, the lack of sequences from additional strains, in particular from the other identified subspecies, has prevented gaining insight into the genomic variability within this species. To fill in this important gap, we undertook the sequencing of a *C. orthopsilosis* clinical isolate assigned to Type 1, a different subspecies than that of the reference strain, and compared both sequences. Our results indicate that the newly sequenced isolate is likely to represent a hybrid between the two *C. orthopsilosis* subspecies. In addition, we find no increase in ploidy and evidence of meiotic recombination between the different haplotypes, strongly suggesting the existence of mating among different *C. orthopsilosis* lineages. Strikingly, this hybrid lineage is represented by at least two clinical isolates from distant continents, suggesting a global spread. In addition, we found several differences in virulence-related gene families between the two strains, pointing to a larger copy number of efflux pumps and secreted lipases in the hybrid. These findings raise the question of the possible role that the formation of hybrids may have in the success and spread of virulent strains. Future analyses are needed to clarify the population structure of this poorly sampled species.

## Materials and Methods

### DNA Extraction

*Candida orthopsilosis* cultures were grown overnight in an orbital shaker (200 rpm, 30 °C) in 2 ml YPD medium (0.5% yeast extract, 1% peptone, and 1% glucose) supplemented with 1% penicillin–streptomycin solution (Sigma). Then, cells were centrifuged (3,000 rpm, 5 min) and were washed twice with 1× sterile PBS. The pellet was resuspended in 500 μl lysis buffer (1 w/V% SDS, 50 mM EDTA, 100 mM Tris pH = 8), 500 μl glass bead was added to the cells, and were disrupted by using a vortex for 3 min; 275 μl 7 M ammonium acetate was added (65 °C, 5 min), and the samples were cooled on ice for 5 min. Then, 500 μl of chloroform–isoamylalcohol (24:1) was added to the mixture, which was then centrifuged for 10 min at 13,000 rpm. The upper phase was transferred to a new microcentrifuge tube, and the previous step was repeated; 500 μl isopropanol was mixed with the upper phase in a new microcentrifuge tube, and the mixture was held in a refrigerator at −20 °C for 5 min. The solution was centrifuged at 13,000 rpm for 10 min. The supernatant was discarded, and the pellet was washed twice with 500 μl 70% ethanol. After the second washing step, the pellet was dried and was resuspended in 100 μl bidistilled water containing RN-ase (Sigma).

### Sporulation Assay

Ascospore formation was investigated under the microscope using various sporulation media (sporulation medium: 0.5% Na acetate, 0.5% KCl, 0.1% yeast extract, 0.05% glucose, 2% agar; Spider medium: 1% mannitol, 1% nutrient broth, 0.2% K_2_HPO_4_, 1.5% agar, at different pH [pH 4, 5, 7, 8, and 9] adjusted with Me Ilvaine buffer solutions; minimal medium supplemented with different C-source: 1% yeast nitrogen base, 2% galactose or 2% maltose or 2% l-sorbose or 2% d-sorbitol or 2% glycerol + 2% agar). The plates were incubated at 25 °C, 30 °C, or at 37 °C with 5% CO_2_ and 100% humidity. The ascospore formation was followed by microscopic observation after 5, 10, 14, 21, and 35 days after inoculation.

### Genome Sequencing

The genome for this strain was obtained at the ultrasequencing core facility of the CRG, using Illumina GAIIx sequencing machine. DNA was fragmented by nebulization or in Covaris to a size approximately 300 bp. After shearing, the ends of DNA fragments were blunted with T4 DNA polymerase and Klenow fragment (New England Biolabs). Then, DNA was purified with a QIAquick polymerase chain reaction (PCR) purification kit (Qiagen). Thereafter, 3′-adenylation was performed by incubation with dATP and 3′–5′-exo-Klenow fragment (New England Biolabs). DNA was purified using MinElute spin columns (Qiagen), and double-stranded Illumina paired-end adapters were ligated to the DNA using rapid T4 DNA ligase (New England Biolabs). After another purification step, adapter-ligated fragments were enriched, and adapters were extended by selective amplification in an 18-cycle PCR reaction using Phusion DNA polymerase (Finnzymes). Libraries were quantified and loaded into Illumina flow cells at concentrations of 7–20 pM. Cluster generation was performed in an Illumina cluster station. Sequence runs of 2 × 76 cycles were performed on the sequencing instrument. Base calling was performed using Illumina pipeline software. In multiplexed libraries, we used 4-bp internal indices (5′-indexed sequences). Deconvolution was performed using the CASAVA software (Illumina).

### Genome Assembly

Reads were preprocessed before assembly to trim at the first undetermined base or at the first base having PHRED quality below 10. The pairs with one (or both) reads shorter than 31 bases after trimming were excluded from the assembly process. SOAPdenovo2 (Luo et al. 2012) was used to assemble paired-ends reads into supercontigs, using K-mer ranging from 31 to 63. As the initial assembly was very fragmented (6,827 contigs) and larger (15.6 Mb) than of the reference strain (12.6 Mb), we assumed the presence of heterozygous regions in our strain (table 1). Heterozygous regions (3 Mb, 4,668 contigs) were successfully removed by Haplomerger version 20120810 (Huang et al. 2012). Subsequently, remaining supercontigs were further scaffolded by SSPACE2 (Boetzer et al. 2011), and gaps were filled using GapCloser from SOAPdenovo package. Finally, supercontigs were scaffolded on *C. orthopsilosis 90-125* chromosomes using Oslay version 1.0 (Richter et al. 2007).

**Table 1:** Genome Sequencing Stats

Sample	Origin	No. Reads (Millions)	Depth of Coverage	Scaffolds	Assembly Size (kb)	GC (%)	Ns	Genes	Homo SNPs	Hetero SNPs
90-125	San Francisco, USA	35.32	235	8	12,659	37.46	177,268	5,700	872	1,184
MCO456	San Antonio, USA	90.22	544	116	13,272	34.42	1,453	5,756	92,497	197,531
AY2	Singapore	—	—	4,152	14,511	37.31	0	—	—	—

Note.—Basic strain and assembly statistics for the genome obtained within this work, the reference strain, and an assembly available in GenBank (AMDC00000000). For each strain the table provides, in this order: Strain name, geographical origin, number of reads obtained, average coverage (× fold), number of scaffolds, total assembly size, GC content, number of bases in gaps, number of predicted genes, and number of homozygous and heterozygous sites. Note that the highly fragmented nature of the AY2 assembly is likely due to the fact that high heterozygosity was not specifically taken care of as we do here.

### Genome Annotation

Genes were predicted using Augustus version 2.5.5 (Stanke et al. 2006) using *C. parapsilosis* CDC317 gene models for training. Predicted gene models were curated using RNA-Seq reads (accession PRJEB5019) by means of exon–intron boundaries and exon skipping. Subsequently, we grouped predicted genes into orthogroups and transferred functional annotation from one-to-one orthologs in model species, that is, *C. albicans* or *Saccharomyces cerevisiae*, based on predictions from the MetaPhOrs approach (Pryszcz et al. 2011). Finally, genes were further annotated using InterProScan 5RC4 (Quevillon et al. 2005).

### Detection of Single-Nucleotide Polymorphisms, Loss of Heterozygosis, and Divergence Estimates

MCO456 reads were aligned onto *C. orthopsilosis 90-125* chromosomes using Bowtie2 with “very sensitive local alignment” mode (Langmead and Salzberg 2012). Single-nucleotide polymorphism (SNPs) and INDELs were called using GATK version 2.1-13 (McKenna et al. 2010). We filtered out clusters of five variants within 20 bases and low-quality variants, as described in GATK documentation (QD < 2.0 || MQ < 40 || FS > 60.0 || HaplotypeScore > 13.0 || MQRankSum < −12.5 || ReadPosRankSum < −8.0). Subsequently, we divided variants into two groups: Homo- and heterozygous. Firstly, we marked parts of MCO456 genome having lower or higher (allowing 25% deviation) coverage than expected as unknown (10%). Then, we defined heterozygous regions as regions having two or more heterozygous sites in a given strain closer than 100 bases. The remaining regions of the genome were considered homozygous, thus the result of loss of heterozygosity (LOH). By analyzing alignments of assembled contig sequences, we initially obtained 99.69% identity between AY2 and MCO456. However, this value may be inflated due to errors in base calls in the assembly. We thus used an alternative strategy consisting in aligning MCO456 reads onto AY2 contigs using BWA MEM version 0.7.5a (Li and Durbin 2010) to call SNPs as previously described. This rendered a higher identity between MCO456 and AY2: 99.9733% (3,369 homozygous SNPs). This should still be considered a minimal estimate because the lack of raw reads for AY2 prevents us from doing a similar correction of that assembly. Assuming similar base call error rate in the two strains, the identity between AY2 and MCO456 should be considered 99.997%. Finally, the distributions of frequencies of read counts at biallelic SNPs were analyzed to estimate ploidy of each chromosome. For this, SNP calls were further filtered ignoring: 1) bases with quality lower than 20, 2) bases in reads with mapping quality lower than 15, and 3) positions with less than 20 reads mapped.

### Detection of Structural Variants

Structural variants were detected using a methodology described and experimentally validated elsewhere (Pryszcz et al. 2013). In brief, variants were first detected using the Delly package (Rausch et al. 2012) and then carefully curated as explained below. In addition, we performed independent analyses based on depth of coverage to detect duplications and deletions not identified by Delly. For every *C. orthopsilosis* gene, we computed number of reads per kilobase of coding sequence per million of aligned reads (RPKM). For any given sequencing experiment, RPKM is expected to be constant unless it is affected by duplication or deletion. We defined a gene being duplicated in a given strain if log 2 of observed versus expected RPKM for that gene was greater than 0.75. Similarly, we called putative deletions if log 2 ratio was smaller than −0.75. Obtained ratios allow not only the detection of duplication/deletions but also inform about the number of copies that are gained or lost. Finally, we used split reads as additional line of evidence. We split in two the reads that were aligned over less than 90% of their length and subsequently realigned these. Alignments created in this way were often flanking putative structural variants. All detected variants were manually curated by visual inspection of the aligned reads. In addition, we generated genome graphs for all chromosomes illustrating copy number variation, and homozygous and heterozygous mutations densities (supplementary file S3, Supplementary Material online). Surprisingly, over half of the identified duplications were shared by *C. orthopsilosis* 90-125 and MCO456. These duplications were not correctly resolved in the reference assembly (90-125) due to the low level of divergence between the two paralogous regions and resulted in assembly gaps (supplementary fig. S1, Supplementary Material online). Inferred assembly gaps are consistently smaller than the nearby duplicated regions, suggesting this was the result of a tandem duplication and pointing to a possible strategy to close these gaps in the reference assembly.

### Phylogenetic Analyses

Concatenated alignment of heterozygous contigs from *C. orthopsilosis* MCO456 and their orthologous regions from *C. orthopsilosis* AY2 and *C. orthopsilosis* 90-125 were generated using Mugsy v1r2.3 (Angiuoli and Salzberg 2011). The resulted alignment consisted of 1,432,069 columns. To root the tree, we performed an additional analysis with the subset of regions that could be aligned to corresponding orthologous regions in *C. parapsilosis* CDC317. This alignment resulted in 632,907 columns. Maximum-likelihood phylogenetic trees were reconstructed using RAxML 7.2.8 using GTRCAT model (Stamatakis et al. 2005).

## Results and Discussion

### Genome Sequencing and Annotation of the MCO456 Strain

Previous work, based on ITS and MAT loci divergence, has established that most *C. orthopsilosis* strains belong to two clearly distinct subspecies, referred to as Type 1 and Type 2 (Sai et al. 2011). The recently sequenced *C. orthopsilosis* reference strain (90-125; from California) belongs to Type 2 (Riccombeni et al. 2012). Here, we sequenced *C. orthopsilosis* strain MCO456 (ATCC96141) originally isolated from a blood sample in San Antonio (TX, USA). This strain can be classified as the alternative Type 1 subspecies based on phylogenetic analyses of the ITS region and *MTL*α idiomorphs (supplementary figs. S2 and S3, Supplementary Material online). The resulting assembly comprises 116 scaffolds and a 544× coverage (table 1; accession PRJEB4430). Automated annotation resulted in 5,756 predicted genes, grouped into 5,279 orthologous groups (including orthologs and in-paralogs), which is roughly similar to the reference strain (table 1). A total of 4,691 orthologous groups are common to *C. parapsilosis* and the two *C. orthopsilosis* strains. Of these, 4,258 are present precisely in one copy in each of these genomes (one-to-one orthologs). The main gene content differences between *C. orthopsilosis* and *C. parapsilosis* have been previously described for the reference strains (Riccombeni et al. 2012). Most such observations are confirmed for the newly sequenced strain. In some cases, the new sequence helps to expand these observations. For instance, the cell-wall assembly protein family Hyr/Iff has been expanded in *C. parapsilosis* relative to *C. orthopsilosis*, although the exact number of copies in the latter could not be determined due to the presence of gaps in the relevant assembly region (Riccombeni et al. 2012). We confirm such finding for the strain sequenced here, in which the absence of assembly gaps in the Hyr/Iff positions allows us to determine the number of copies to 4, when compared with 17 in *C. parapsilosis*. Most of the differences in gene content correspond to differences in copy number within gene families and others that can be attributable to differences in annotation or coverage between the two assemblies (supplementary table S1, Supplementary Material online). We focused on differences between the two strains that are supported by reciprocal mapping of raw reads and are therefore independent of differences in the assembly and/or the annotation procedures. Importantly, several of these well-supported differences correspond to virulence-related gene families (see below).

### Structural and Gene Content Variations between 90-125 and MCO456 Strains

**Fig. 1.—:**
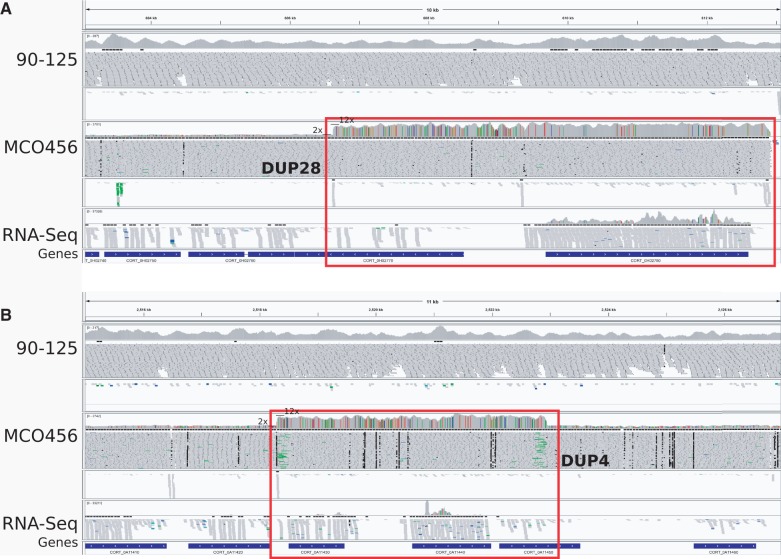
Copy number differences between *Candida orthopsilosis* strains. Two duplications, DUP28 (*A*) and DUP4 (*B*), were analyzed in detail (marked with red rectangles). For each duplication, ten panels are given in this order, from top to down: Genomic coordinates; genomic coverage, genomic reads alignments, and split-read mapping for 90-125; genomic coverage, genomic reads alignments, and split-read mapping for MCO456; transcriptomic coverage and transcriptomic reads alignments from MCO456; and predicted loci. Both regions, DUP28 and DUP4, are fully homozygous and present in 12 copies in the genome when compared with two in *C. orthopsilosis* 90-125 reference genome. All copies of DUP4 are most likely in tandem, whereas copies of DUP28 are spread through at least five chromosomes. A genes encoded within DUP28 (CORT_0H02780, which encodes a putative outer membrane Na/K efflux pump) and DUP4 (CORT_0A11440, which encodes a vacuolar transporter) are expressed at higher levels, when compared with their neighboring genes.

Using a remapping strategy, we identified structural variants in both sequenced *C. orthopsilosis* strains (see Materials and Methods). Altogether, we identified 29 duplications (supplementary table S2, Supplementary Material online) and 103 deletions (supplementary table S3, Supplementary Material online). Over half of the identified duplications (17) were shared by *C. orthopsilosis* 90-125 and MCO456 and correspond to collapsed regions in the published assembly of 90-125. By using pair-end mapping information, we could infer that at least 17 out of the 29 detected duplications were intrachromosomal and that the additional copy is likely to be placed several kilobase up- or downstream, coinciding with nearby gaps in the reference assembly (Materials and Methods and supplementary fig. S1, Supplementary Material online). The largest duplication (DUP2, a segmental duplication of 238 kb) was found in *C. orthopsilosis 90-125* and represents a collapsed region in the reference assembly (fig. 1 and supplementary fig. S4, Supplementary Material online). This duplication is located at chromosome HE681719 and affects 74 genes (supplementary table S4, Supplementary Material online). Besides the rRNA cluster (DUP29), the gene expansion involving the highest number of paralogous copies (5–6×, DUP28) was found in *C. orthopsilosis MCO456* and affected the gene *CORT_0H02780*, which encodes a putative outer membrane Na/K efflux pump. The resulting approximately 12 copies of the gene—when compared with just two copies in 90-125—are distributed over five chromosomes and are all identical at the nucleotide level, pointing to a recent expansion. A large fraction of the identified deletions (85 out of 103) are homozygous, 43 affect coding genes and 5 result in putative gene fusions. Similar to what has been reported for *C. parapsilosis* (Pryszcz et al. 2013), a significant number of deletions (40) was surrounded by DNA direct repeats, suggesting single-strand annealing as the prevalent mechanism of DNA double-strand break repair in *C. orthopsilosis* (Ivanov et al. 1996). The largest deletion (DEL51) is 7,300-bp long and is surrounded by 824 bases long direct repeats. The two genes flanking this deletion (*CORT_0C02740* and *CORT_0C02745*) are both co-orthologous to *C. albicans* Iff5, a GPI-anchored, adhesin-like protein. Interestingly, Iff5 has been expanded in *C. orthopsilosis* 90-125 and *C. parapsilosis*, but *C. orthopsilosis* MCO456 encodes only one copy due to this deletion.

A total of 446 gene families differ in the number of members between any of *C. orthopsilosis* strains or *C. parapsilosis* (supplementary table S1, Supplementary Material online). Of these, 239 contain more members in MCO456 than in 90-125, whereas only 56 contain more members in 90-125 than in MCO456. Notably, Iff5 seems to be the only virulence-related gene family that is present in a higher number of copies in 90-125 strain, when compared with MCO456. For several other virulence-related gene families, the newly sequenced strain presented lineage-specific expansions. For instance, MCO456 shows a significantly larger number of efflux pumps from the major facilitator superfamily (MFS) with 142 MFS-coding genes distributed across 83 different MFS clades, when compared with 128 in 90-125 (supplementary table S1, Supplementary Material online). Thus, MCO456 efflux-pump toolkit is closer to that of *C. parapsilosis* (147 members). Similarly, *C. orthopsilosis* MCO456 have two additional secreted aspartic proteases (SAPs), which have been associated with *C. albicans* and *C. parapsilosis* virulence, through their involvement in the degradation of components of the innate immune system and survival in macrophages (Naglik et al. 2003; Horváth et al. 2012), with 25 SAP-coding genes, when compared with 23 in 90-125 and 26 in *C. parapsilosis* (supplementary table S1, Supplementary Material online). Finally, *C. orthopsilosis* MCO456 strain displays a specific duplication of a cluster of lipases resulting in four members, when compared with two in 90-125 and *C. parapsilosis* (supplementary table S1, Supplementary Material online, cluster #30). This finding is particularly interesting, because secreted fungal lipases are thought to have a role in many virulence associated processes, including nutrient acquisition by liberation of free fatty acids from lipid molecules, adhesion to host cells, and generation of inflammatory mediators, and differences in the ability to secrete lipases among *C. orthopsilosis* strains have been noted (Gácser, Stehr, et al. 2007). Although the actual relevance to virulence properties of these genomic differences remains to be established, it is nevertheless remarkable that several of the few differences found between the strains consistently hint toward an expanded virulence toolkit in MCO456. Notably, we have previously investigated the virulence of the MCO456 strain in vitro, along with other *C. orthopsilosis* isolates (Németh et al. 2013). It forms pseudohyphae and produces extracellular proteinases but lacks extracellular lipase activity, similarly to other *C. orthopsilosis* strains. Furthermore, the phagocytosis of MCO456 strain by J774.2 mouse macrophages was comparable to the phagocytosis of *C. parapsilosis* sensu stricto. Similarly, some of the differences in gene content hint to families involved in drug resistance (see supplementary table S1, Supplementary Material online). These include six more members in MCO456, than in 90-125, of the amino acid permease DIP5 involved in resistance to ergosterol analogs in *C. albicans* (Xu et al. 2007), six more members of the Pleiotropic drug resistance subfamily of ABC transporters, and three more members of the MFS transporters and of homologs of SEC7, all of the families related to “response to drug” processes in *C. albicans*, according to Candida Genome Database (Binkley et al. 2014). Finally, MCO456 carry five mutations in *ERG3* (*CORT_0E05900*), a gene frequently found mutated in *C. albicans* strains resistant to azoles (Martel et al. 2010), including two affecting the promoter and one nonsynonymous mutation (Y13C). Again, assessing whether these genomic differences effectively confer some increased resistance to antifungal drugs needs to be established experimentally. Susceptibility to some drugs has only been studied in MCO456 in a comparative analysis with other *C. parapsilosis* complex species (Szenzenstein et al. 2013). In that study, MIC_100_ value in liquid medium (100% growth inhibition) of atorvastatin was 25 mg/ml for *C. parapsilosis* and 50 mg/ml for *C. orthopsilosis* (MCO456). *Candida orthopsilosis* MCO456 was found to be the less susceptible to fluvastatin when compared with *C. parapsilosis* and *C. metapsilosis*, as 12.5 mg/ml concentration caused only 50% growth inhibition. Direct comparison of the two sequenced strains in terms of virulence and antifungal resistance properties is needed to establish whether the hybridization is associated to changes in these phenotypes.

### *Candida orthopsilosis* MCO456 Is a Hybrid between Two Distinct Subspecies

**Fig. 2.—:**
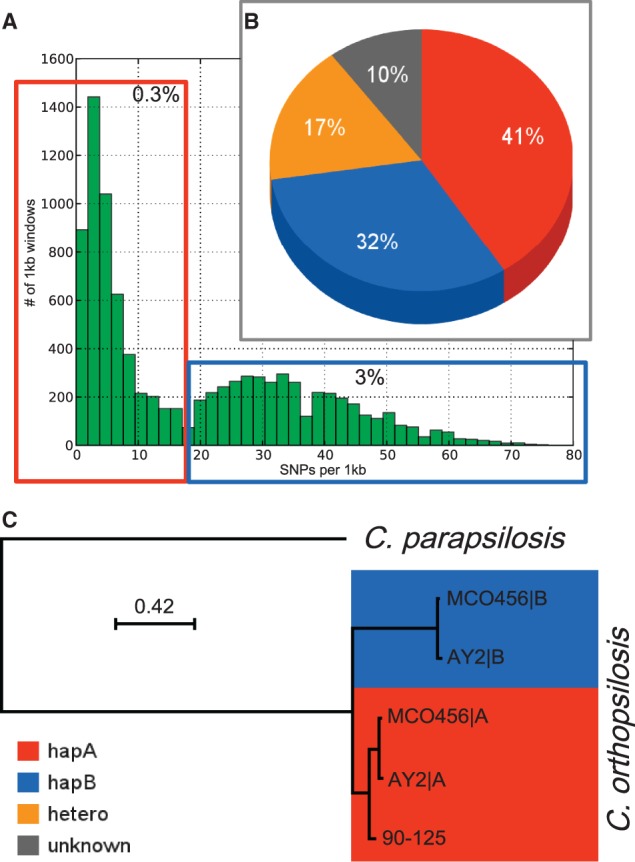
*Candida orthopsilosis* MCO456 is a hybrid of two subspecies. Distribution of number of SNPs between 1 kb homozygous regions of MCO456 and 90-125 is bimodal, with peaks around 0.3% and above 3% interstrain divergence (*A*). The genome of MCO456 can be assigned into four categories: Haplotype A (close in sequence to 90-125, therefore Type 2), haplotype B (Type 1), heterozygous, and unknown (*B*). The distance between the two haplotypes in heterozygous regions of MCO456 and AY2 is larger than distances between strains but smaller than distance to the closest species; therefore, we hypothesize that MCO456 is hybrid of two *C. orthopsilosis* subspecies (*C*).

The most striking observed difference between the two strains, however, was the high degree of heterozygosity found in MCO456 with a total of 2,182 kb heterozygous sites, representing 17% of the genome, when compared with the very low fraction of heterozygous sites in 90-125 (12.6 kb, 0.1%). The heterozygous sites in MCO456 are clustered in 4,872 regions that show an average interallelic divergence of 4.5%. Interestingly, the distribution of distances to 90-125 in MCO456 homozygous regions is bimodal (fig. 2*A*): 5,169 kb (41%) of the genome is nearly identical to 90-125 genome (0–18 SNPs per 1 kb, this is ≤1.8% divergence) with an average divergence of 1.12% and 4.015 kb (32%) is diverged above 1.8% (more than 18 SNPs per 1 kb) with an average divergence to 90-125 of 7.63% (supplementary file S3, Supplementary Material online). These results indicate that *C. orthopsilosis* MCO456 is a hybrid between two parental lineages, one of which being very close to Type 2 strains such as 90-125. The other haplotype, which contains the ITS region and the MAT locus, would correspond to a parental that would have been classified as Type 1 subspecies (supplementary figs. S2 and S3, Supplementary Material online). In fact, the MTL locus of MCO456 has an α idiomorph and is 100% identical to Cp289 Type 1 strain. In contrast, this locus has 95.34% divergence to the α idiomorph of Type 2 strains (represented by strain Cp185); 90-125 is an MTLa idiomorph. On the other hand, rDNA clusters of 90-125 and MCO456 are both homozygous and very similar in sequence, pointing to an origin of the rDNA in cluster in the hybrid from the Type 2 parental (supplementary file S3, Supplementary Material online). To estimate ploidy level in the putative hybrid, we investigated the distribution of read counts at biallelic SNPs for each mapped chromosomes. In a diploid genome, the mean of read counts at heterozygous positions should have a single mode around 0.5, whereas we would expect two modes, 0.33 and 0.67, for triploids, and three modes, 0.25, 0.5, and 0.75 for tetraploids (Yoshida et al. 2013). Our results revealed a single peak around 0.5, indicating MCO456 is a diploid and no sign for large-scale aneuploidy is present in any of the chromosomes (supplementary fig. S5, Supplementary Material online).

Interestingly, mitochondrial genomes of both strains are homogeneous, with no sequence variation detected even at the high resolution employed (>5,600× sequence coverage of the mitochondrial genome). In addition, the mitochondrial sequences from both strains are very similar in sequence, with 70–77 SNPs in the entire mitochondrial genome, corresponding to 0.3% divergence. This indicates that the mitochondrion from MCO456 was entirely inherited from the Type 2 parental. This result is consistent with uniparental mitochondrion inheritance within *Candida* spp. (Ni et al. 2011), although previous observations of chimeric sequences in *C. orthopsilosis* mitochondrial genomes (Valach et al. 2012) suggest that recombination could also occur between the two parental mitochondrial genomes. Altogether our results point to a chimeric genome of MCO456 with two distinct haplotypes at approximately 5% divergence. One of these two haplotypes contains a MAT locus identical to that of Type I strains, whereas the rDNA locus and the mitochondrial genome are nearly identical to those in Type II strains.

Admittedly, the lack of a fully sequenced, homozygous Type 1 genome prevents us from fully describing one of the parentals. However, alternative hypotheses for the formation of this architecture that do not involve the hybridization of two genomes differing by approximately 5% seem less plausible and are not compatible with the data presented. For instance, a whole-genome duplication followed by divergence is not consistent with the lack of large blocks of conserved synteny, as detected in *S. cerevisiae*, or with a finding of a higher similarity of one of the haplotypes and the mitochondrial genome with another strain. Similarly, one could think of 90-125 as a derivative, by massive loss of heterozygosis from a highly heterozygous strain, but this strain has a different MTL idiomorph. Furthermore, the existence of loss of massive heterozygosity in one strain does not explain the origin of heterozygosity in the first place and, considering the divergence detected, would invoke massive LOH in a very short time span. Finally, we investigated the sporulation ability of the hybrid strain on various media, pH, temperature, and incubation time (see Materials and Methods). Microscopic observation revealed no ascospore formation in the cultures, thus no sporulation could be induced in the hybrid strain under the conditions we tested. However, considering the difficulty of studying this process in *Candida* spp. (Sai et al. 2011), these results should not be considered definitive.

In its present evolutionary state, 17% of the sequenced hybrid genome for which we can assign a haplotype has conserved heterozygous regions from its two parentals, whereas 32% and 41% of the genome were inherited from either the Type 1 or Type 2 parental, respectively (fig. 2*B*). Of note, this roughly balanced distribution of genomic regions inherited from both parentals contrasts with a previously reported interspecies hybrid within the “CTG” clade, that of *Pichia* (*Meyerozyma*) *sorbitophila*, where a high unbalance between both parentals (36–5%) was found (Louis et al. 2012). This difference may be explained by the lower genetic divergence between parentals in the *C. orthopsilosis* case (5% when compared with 15% in *M. sorbitophila*), which will result in a lower risk of genetic incompatibility (Bateson–Dobzhansky–Muller effect) (Cutter 2012). In such a case, LOH toward any of the parental haplotypes would be expected to evolve nearly neutrally. Consistent with this idea, we did not find any functional enrichment among genes present in each of the three genotypic categories. Furthermore, considering that we did not find large deletions, the erosion of the differences between parental chromosomes seems mainly due to homologous recombination between the two haplotypes initially present in distinct homologous chromosomes. Consistent with this, we identified numerous genome regions where the existence of past recombination events is supported at the sequence level (see supplementary file S4, Supplementary Material online). Repeated recombination between homologous chromosomes would result in the current observed pattern with extense regions of loss of heterozygosity and different haplotypes present in the same chromosome. Recombination can occur in both meiotic (sexual recombination) and mitotic cells, but they leave different genomic signatures that can be recognized with resequencing data. Indeed, in the case of sexual recombination, we expect chromosome size to have a negative correlation with the length of LOH tracks and a positive correlation with levels of heterozygosis, because recombination rate per basepair increases with chromosome size (Yin and Petes 2013). In contrast, mitotic recombination will leave the opposite trend. Our results (supplementary fig. S6, Supplementary Material online) show a negative correlation of the length of LOH tracks with chromosome size (Spearman’s *r* = −0.76, *P* < 0.05) and a positive correlation of the fraction of heterozygous sites with chromosome size (Spearman’s *r* = 0.71, *P* < 0.05). Thus, our results suggest a significant role of meiotic recombination in shaping the current hybrid structure and provide evidence for the existence of sexual recombination in *C. orthopsilosis.*

### Global Spread and Virulence of Types 1–2 Hybrid Lineage

We sought further evidence of hybrid strains in *C. orthopsilosis* by inspecting the available genomic sequence of AY2 strain, isolated from a persistent skin infection in Singapore (Chan et al. 2011), Genbank AMDC00000000). Strikingly, this strain was found to represent a very similar hybrid to MCO456, with regions of homozygosity and heterozygosity showing a high level of overlap and limited divergence at the sequence level between AY2 and MCO456 (99.997% identity at the nucleotide level). This indicates that these geographically distant clinical isolates are highly related and derive from the same ancestral hybridization event. It remains to be established whether such hybridization event may have facilitated the persistence and spread of this clade with demonstrated virulence. We reconstructed the phylogenetic relationships between all *C. orthopsilosis* haplotypes by using 1,432,069 sites in common heterozygous regions in MCO456 and AY2 (fig. 2*C*). With the current genomic sampling, we cannot determine the exact origin of the hybrid lineage, which should have happened somewhere before the separation of MCO456/AY2 and after their divergence from the 90-125 lineage. Note that the level of sequence divergence between MCO456 and AY2 is too close to the error rate of current sequencing technologies to properly estimate their time of divergence. Moreover, we found that regions of heterozygosity were almost completely overlapping with minor differences possible due to assembly errors. It is remarkable that two independent isolates from the same hybrid lineages have been found in opposite sides of the globe and that they are both related to infection episodes. This indicates that the hybrid line is virulent and that it has spread.

## Concluding Remarks

We investigated the extent of genome-wide divergence among two recognized subspecies of *C. orthopsilosis*, by sequencing an isolate classified as Type 1 subspecies and comparing it with the Type 2 sequenced isolate. Unexpectedly, we found that the selected isolate was a hybrid of the two subspecies under study. This conclusion is strongly supported by the presence of a highly heterozygous genome in which one of the haplotypes is largely similar to the Type 1 sequenced isolate, whereas the other haplotype, which contains the Type-1 defining MAT and ITS loci, has an average divergence of 5%. In addition, the hybrid genome was found to be diploid and showing patterns of heterozygosity compatible with the effect of meiotic recombination. This discovery suggests the existence of mating between distinct *C. orthopsilosis* lineages, supporting previous indications (Tavanti et al. 2007), and arguing against recent speculations of a lack of recombination within *C. orthopsilosis* (Sai et al. 2011; Riccombeni et al. 2012). Furthermore, the hybrid originates from two relatively distant lineages, which underscores the importance of hybridization as an evolutionary mechanism having a role in the *Candida* clade. Considering that cells from *Candida* species respond to mating pheromones of different species within the clade, our results anticipate the possibility of finding more hybrids within this pathogenic group of species. These mechanisms can have important implications in how relevant virulence phenotypes such as adhesion capabilities or drug resistance could evolve within pathogenic lineages. Hybridization has been recently recognized as an important and widespread process in fungi, but its relevance for pathogenesis remains largely unexplored (Xu et al. 2002; González et al. 2006; Morales and Dujon 2012). Finally, we show that two clinical strains isolated in very distant geographical locations (Texas and Singapore) are close representatives of the same hybrid lineage. This demonstrates that the hybrid lineage is pathogenic and distributed globally. The significance of this finding—two of the three clinical independent (and distant) isolates are from the same hybrid lineage—raises the question of whether hybridization and virulence properties are related. It is tempting to speculate that hybridization may have conferred some sort of selective advantage in the environment that have facilitated its spread, whereas additionally conferring increased virulence properties. We indeed detected that the hybrid presents higher copy numbers in genes related to pathogenesis such as efflux pumps or secreted lipases. Conversely, one may wonder whether the stressful conditions in the host may have facilitated the formation of hybrids. Hybridization can trigger the formation of new species that can display unique phenotypic features, and this mechanism has been proposed to play a role in the emergence of globally spread virulent lineages in other fungal pathogens (Stukenbrock et al. 2012). Certainly, our current genomic sampling and limited availability of population and phenotypic data prevent us from providing definitive answers to many of these questions. Further research is thus needed to address these questions, including the direct comparison of virulence and antifungal resistance of the two sequenced strains.

## Supplementary Material

Supplementary files S1–S4 are available at *Genome Biology and Evolution* online (http://www.gbe.oxfordjournals.org/).

Supplementary Data
