# Supplementary material for: Genome Comparison of Candida orthopsilosis Clinical Strains Reveals the Existence of Hybrids between Two Distinct Subspecies
Source: Genome Biol Evol. 2014 Apr 18;6(5):1069–78. doi: 10.1093/gbe/evu082 (PMC4040990; doi:10.1093/gbe/evu082)
Supplement: Supplementary Data [file supp_6_5_1069__index.html]

Genome comparison of Candida orthopsilosis clinical strains reveals the existence of hybrids between two distinct subspecies — Genome Comparison of Candida orthopsilosis Clinical Strains Reveals the Existence of Hybrids between Two Distinct Subspecies — Supplementary Data 

# Genome Comparison of *Candida orthopsilosis* Clinical Strains Reveals the Existence of Hybrids between Two Distinct Subspecies

## Supplementary Data

files

**Files in this Data Supplement:**

- Supplementary Data - pdf file
- Supplementary Data - pdf file
- Supplementary Data - pdf file
- Supplementary Data - xls file
