# Supplementary material for: Genome Comparison of Candida orthopsilosis Clinical Strains Reveals the Existence of Hybrids between Two Distinct Subspecies
Source: Genome Biol Evol. 2014 Apr 18;6(5):1069–78. doi: 10.1093/gbe/evu082 (PMC4040990; doi:10.1093/gbe/evu082)
Supplement: Supplementary Data [file supp_evu082_SuppFile1.pdf]

Supplementary figures

Supplementary figure S1: Gaps in the reference chromosomes...2
Supplementary figure S2: Alignment of the ITS region of rDNA from C. parapsilosis, C. orthopsilosis and C. metapsilosis...3
Supplementary figure S3: Mating type locus in C. orthopsilosis...6
Supplementary figure S4: The largest identified duplication (DUP2)...8
Supplementary figure S5: Ploidy analyses...9
Supplementary figure S6: C. orthopsilosis MCO456 chromosomes characteristics.........................10
References..11

Supplementary figure S1: Gaps in the reference chromosomes

Three duplications common to 90-125 and MCO456 were investigated in detail: DUP8 (A), DUP18 (B) and DUP25 (C). Gaps in reference chromosomes can be explained by collapsed duplications that are present several kb downstream. These duplications were not correctly recognised during assembly process as such due to no or little divergence between two copies in 90-125. Split-reads mapping (blue and green horizontal lines) supports this hypothesis: part of the read align at gap end, while the other part at duplication end.

Duplications are marked with red rectangles, while gaps under discussion are denoted with red arrows. For each duplication ten panels are given: coordinate; genomic coverage, reads alignments and split-read mapping for 90-125; genomic coverage, reads alignments and split-read mapping for MCO456; transcriptomic coverage and reads alignments from MCO456; and predicted loci.

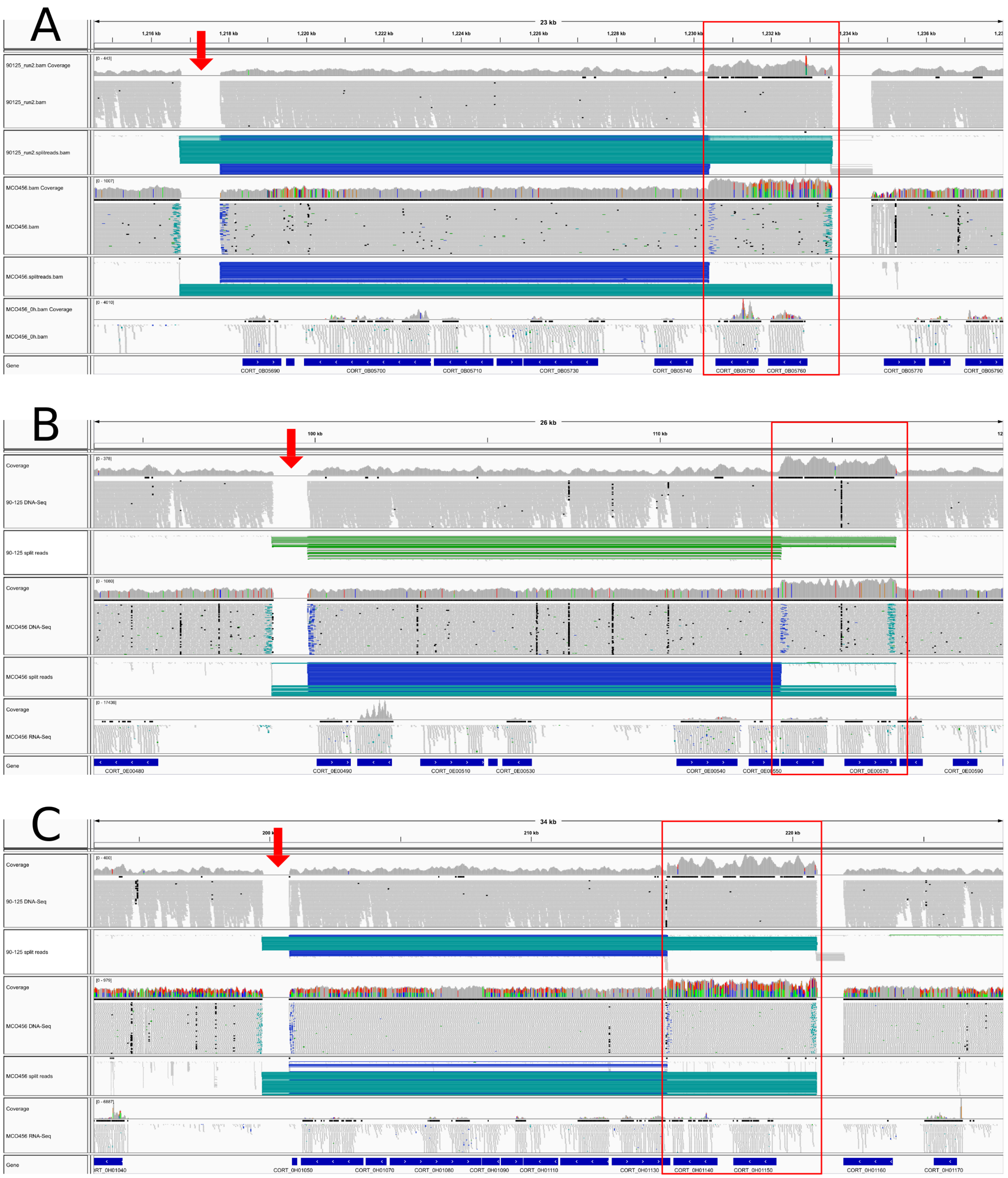

Supplementary figure S2: Alignment of the ITS region of rDNA from *C. parapsilosis, C. orthopsilosis and C. metapsilosis*

The ITS sequence from MCO456 and AY2 were obtained from genome projects: GenBank accessions: PRJEB4430 and PRJNA171019. The remaining ITS regions were previously published (Sai et al., 2011). The alignment was prepared using Muscle v3.8.31 (Edgar, 2004).

C_met: C. metapsilosis; C_ort: C. orthopsilosis; C_par: C. parapsilosis. Type 1 and Type 2 isolates of C. orthopsilosis are highlighted in yellow and green, respectively. MTLa and MTLα are highlighted in grey and red, respectively. The differences in sequence of the ITS region are also indicated. The ITS region of C. orthopsilosis 90-125 is intermediate between the Type 1 and Type 2 isolates, while the ITS region of MCO456 is identical to Type 2 isolates.

```
C_par_AY391843         TTGGAGTTTGTACCAATGAGTGG----AAAAAACCTATCCATTAGTTTATACTCCGCCTT
C_met_ATCC96143        TTGGAGTTTGTACCAATGAGTGGAAAAAAAAAACCTATCCATTAGTTTATACTCCGCCTT
C_met_ATCC96144T       TTGGAGTTTGTACCAATGAGTGGAAAAAAAAAACCTATCCATTAGTTTATACTCCGCCTT
C_met_Cp43             TTGGAGTTTGTACCAATGAGTGGAAAAAAAAAACCTATCCATTAGTTTATACTCCGCCTT
C_ort_T1_MCO456_α      TTGGAGTTTGTACCAATGAGTGG---AAAAAAACCTATCCATTAGTTTATACTCCGCCTT
C_ort_AY2_α            TTGGAGTTTGTACCAATGAGTGG---AAAAAAACCTATCCATTAGTTTATACTCCGCCTT
C_ort_T1_Cp344_a       TTGGAGTTTGTACCAATGAGTGG---AAAAAAACCTATCCATTAGTTTATACTCCGCCTT
C_ort_T1_Cp289_α       TTGGAGTTTGTACCAATGAGTGG---AAAAAAACCTATCCATTAGTTTATACTCCGCCTT
C_ort_T1_Co288_a       TTGGAGTTTGTACCAATGAGTGG---AAAAAAACCTATCCATTAGTTTATACTCCGCCTT
C_ort_T1_Cp85_α        TTGGAGTTTGTACCAATGAGTGG---AAAAAAACCTATCCATTAGTTTATACTCCGCCTT
C_ort_T1_Cp47_a        TTGGAGTTTGTACCAATGAGTGG---AAAAAAACCTATCCATTAGTTTATACTCCGCCTT
C_ort_T1_Cp25_a        TTGGAGTTTGTACCAATGAGTGG---AAAAAAACCTATCCATTAGTTTATACTCCGCCTT
C_ort_T1_942211K_a     TTGGAGTTTGTACCAATGAGTGG---AAAAAAACCTATCCATTAGTTTATACTCCGCCTT
C_ort_ATCC96139T       TTGGAGTTTGTACCAATGAGTGG---AAAAAAACCTATCCATTAGTTTATACTCCGCCTT
C_ort_T2_Cp185_α       TTGGAGTTTGTACCAATGAGTGG--AAAAAAAACCTATCCATTAGTTTATACTCCGCCTT
C_ort_T2_Cp331_α       TTGGAGTTTGTACCAATGAGTGG--AAAAAAAACCTATCCATTAGTTTATACTCCGCCTT
C_ort_T2_Cp296_a       TTGGAGTTTGTACCAATGAGTGG--AAAAAAAACCTATCCATTAGTTTATACTCCGCCTT
C_ort_T2_Cp269_a       TTGGAGTTTGTACCAATGAGTGG--AAAAAAAACCTATCCATTAGTTTATACTCCGCCTT
C_ort_T2_Cp125_a/α     TTGGAGTTTGTACCAATGAGTGG--AAAAAAAACCTATCCATTAGTTTATACTCCGCCTT
C_ort_T2_90_125_a      TTGGAGTTTGTACCAATGAGTGG---AAAAAAACCTATCCATTAGTTTATACTCCGCCTT
                       ***********************    *********************************

C_par_AY391843         TCTTTCAAGCAAACCCAGCGTATCGCTCAACACCAAACCCGAGGGTTTGAGGGAGAAATG
C_met_ATCC96143        TCTTTCAAGCAAACCCAGCGTATCGCTCAACACCAAACCCGAAGGTTTGAGGGAGAAATG
C_met_ATCC96144T       TCTTTCAAGCAAACCCAGCGTATCGCTCAACACCAAACCCGAAGGTTTGAGGGAGAAATG
C_met_Cp43             TCTTTCAAGCAAACCCAGCGTATCGCTCAACACCAAACCCGAAGGTTTGAGGGAGAAATG
C_ort_T1_MCO456_α      TCTTTCAAGCAAACCCAGCGTATCGCTCAACACCAAACCCGAAGGTTTGAGGGAGAAATG
C_ort_AY2_α            TCTTTCAAGCAAACCCAGCGTATCGCTCAACACCAAACCCGAAGGTTTGAGGGAGAAATG
C_ort_T1_Cp344_a       TCTTTCAAGCAAACCCAGCGTATCGCTCAACACCAAACCCGAAGGTTTGAGGGAGAAATG
C_ort_T1_Cp289_α       TCTTTCAAGCAAACCCAGCGTATCGCTCAACACCAAACCCGAAGGTTTGAGGGAGAAATG
C_ort_T1_Co288_a       TCTTTCAAGCAAACCCAGCGTATCGCTCAACACCAAACCCGAAGGTTTGAGGGAGAAATG
C_ort_T1_Cp85_α        TCTTTCAAGCAAACCCAGCGTATCGCTCAACACCAAACCCGAAGGTTTGAGGGAGAAATG
C_ort_T1_Cp47_a        TCTTTCAAGCAAACCCAGCGTATCGCTCAACACCAAACCCGAAGGTTTGAGGGAGAAATG
C_ort_T1_Cp25_a        TCTTTCAAGCAAACCCAGCGTATCGCTCAACACCAAACCCGAAGGTTTGAGGGAGAAATG
C_ort_T1_942211K_a     TCTTTCAAGCAAACCCAGCGTATCGCTCAACACCAAACCCGAAGGTTTGAGGGAGAAATG
C_ort_ATCC96139T       TCTTTCAAGCAAACCCAGCGTATCGCTCAACACCAAACCCGAAGGTTTGAGGGAGAAATG
C_ort_T2_Cp185_α       TCTTTCAAGCAAACCCAGCGTATCGCTCAACACCAAACCCGAAGGTTTGAGGGAGAAATG
C_ort_T2_Cp331_α       TCTTTCAAGCAAACCCAGCGTATCGCTCAACACCAAACCCGAAGGTTTGAGGGAGAAATG
C_ort_T2_Cp296_a       TCTTTCAAGCAAACCCAGCGTATCGCTCAACACCAAACCCGAAGGTTTGAGGGAGAAATG
C_ort_T2_Cp269_a       TCTTTCAAGCAAACCCAGCGTATCGCTCAACACCAAACCCGAAGGTTTGAGGGAGAAATG
C_ort_T2_Cp125_a/α     TCTTTCAAGCAAACCCAGCGTATCGCTCAACACCAAACCCGAAGGTTTGAGGGAGAAATG
C_ort_T2_90_125_a      TCTTTCAAGCAAACCCAGCGTATCGCTCAACACCAAACCCGAAGGTTTGAGGGAGAAATG
                       ****************************************** *****************
```

```
C_par_AY391843         ACGCTCAAACAGGCATGCCCTTTGGAATACCAAAGGGCGCAATGTGCGTTCAAAGATTCG
C_met_ATCC96143        ACGCTCAAACAGGCATGCCCTTTGGAATACCAAAGGGCGCAATGTGCGTTCAAAGATTCG
C_met_ATCC96144T       ACGCTCAAACAGGCATGCCCTTTGGAATACCAAAGGGCGCAATGTGCGTTCAAAGATTCG
C_met_Cp43             ACGCTCAAACAGGCATGCCCTTTGGAATACCAAAGGGCGCAATGTGCGTTCAAAGATTCG
C_ort_T1_MC0456_α      ACGCTCAAACAGGCATGCCCTTTGGAATACCAAAGGGCGCAATGTGCGTTCAAAGATTCG
C_ort_AY2_α            ACGCTCAAACAGGCATGCCCTTTGGAATACCAAAGGGCGCAATGTGCGTTCAAAGATTCG
C_ort_T1_Cp344_a       ACGCTCAAACAGGCATGCCCTTTGGAATACCAAAGGGCGCAATGTGCGTTCAAAGATTCG
C_ort_T1_Cp289_α       ACGCTCAAACAGGCATGCCCTTTGGAATACCAAAGGGCGCAATGTGCGTTCAAAGATTCG
C_ort_T1_Co288_a       ACGCTCAAACAGGCATGCCCTTTGGAATACCAAAGGGCGCAATGTGCGTTCAAAGATTCG
C_ort_T1_Cp85_α        ACGCTCAAACAGGCATGCCCTTTGGAATACCAAAGGGCGCAATGTGCGTTCAAAGATTCG
C_ort_T1_Cp47_a        ACGCTCAAACAGGCATGCCCTTTGGAATACCAAAGGGCGCAATGTGCGTTCAAAGATTCG
C_ort_T1_Cp25_a        ACGCTCAAACAGGCATGCCCTTTGGAATACCAAAGGGCGCAATGTGCGTTCAAAGATTCG
C_ort_T1_942211K_a     ACGCTCAAACAGGCATGCCCTTTGGAATACCAAAGGGCGCAATGTGCGTTCAAAGATTCG
C_ort_ATCC96139T       ACGCTCAAACAGGCATGCCCTTTGGAATACCAAAGGGCGCAATGTGCGTTCAAAGATTCG
C_ort_T2_Cp185_α       ACGCTCAAACAGGCATGCCCTTTGGAATACCAAAGGGCGCAATGTGCGTTCAAAGATTCG
C_ort_T2_Cp331_α       ACGCTCAAACAGGCATGCCCTTTGGAATACCAAAGGGCGCAATGTGCGTTCAAAGATTCG
C_ort_T2_Cp296_a       ACGCTCAAACAGGCATGCCCTTTGGAATACCAAAGGGCGCAATGTGCGTTCAAAGATTCG
C_ort_T2_Cp269_a       ACGCTCAAACAGGCATGCCCTTTGGAATACCAAAGGGCGCAATGTGCGTTCAAAGATTCG
C_ort_T2_Cp125_a/α     ACGCTCAAACAGGCATGCCCTTTGGAATACCAAAGGGCGCAATGTGCGTTCAAAGATTCG
C_ort_T2_90_125_a      ACGCTCAAACAGGCATGCCCTTTGGAATACCAAAGGGCGCAATGTGCGTTCAAAGATTCG
                       ************************************************************

C_par_AY391843         ATGATTCACGAATATCTGCAATTCATATTACTTATCGCATTTCGCTGCGTTCTTCATCGA
C_met_ATCC96143        ATGATTCACGAATATCTGCAATTCATATTACTTATCGCATTTCGCTGCGTTCTTCATCGA
C_met_ATCC96144T       ATGATTCACGAATATCTGCAATTCATATTACTTATCGCATTTCGCTGCGTTCTTCATCGA
C_met_Cp43             ATGATTCACGAATATCTGCAATTCATATTACTTATCGCATTTCGCTGCGTTCTTCATCGA
C_ort_T1_MC0456_α      ATGATTCACGAATATCTGCAATTCATATTACTTATCGCATTTCGCTGCGTTCTTCATCGA
C_ort_AY2_α            ATGATTCACGAATATCTGCAATTCATATTACTTATCGCATTTCGCTGCGTTCTTCATCGA
C_ort_T1_Cp344_a       ATGATTCACGAATATCTGCAATTCATATTACTTATCGCATTTCGCTGCGTTCTTCATCGA
C_ort_T1_Cp289_α       ATGATTCACGAATATCTGCAATTCATATTACTTATCGCATTTCGCTGCGTTCTTCATCGA
C_ort_T1_Co288_a       ATGATTCACGAATATCTGCAATTCATATTACTTATCGCATTTCGCTGCGTTCTTCATCGA
C_ort_T1_Cp85_α        ATGATTCACGAATATCTGCAATTCATATTACTTATCGCATTTCGCTGCGTTCTTCATCGA
C_ort_T1_Cp47_a        ATGATTCACGAATATCTGCAATTCATATTACTTATCGCATTTCGCTGCGTTCTTCATCGA
C_ort_T1_Cp25_a        ATGATTCACGAATATCTGCAATTCATATTACTTATCGCATTTCGCTGCGTTCTTCATCGA
C_ort_T1_942211K_a     ATGATTCACGAATATCTGCAATTCATATTACTTATCGCATTTCGCTGCGTTCTTCATCGA
C_ort_ATCC96139T       ATGATTCACGAATATCTGCAATTCATATTACTTATCGCATTTCGCTGCGTTCTTCATCGA
C_ort_T2_Cp185_α       ATGATTCACGAATATCTGCAATTCATATTACTTATCGCATTTCGCTGCGTTCTTCATCGA
C_ort_T2_Cp331_α       ATGATTCACGAATATCTGCAATTCATATTACTTATCGCATTTCGCTGCGTTCTTCATCGA
C_ort_T2_Cp296_a       ATGATTCACGAATATCTGCAATTCATATTACTTATCGCATTTCGCTGCGTTCTTCATCGA
C_ort_T2_Cp269_a       ATGATTCACGAATATCTGCAATTCATATTACTTATCGCATTTCGCTGCGTTCTTCATCGA
C_ort_T2_Cp125_a/α     ATGATTCACGAATATCTGCAATTCATATTACTTATCGCATTTCGCTGCGTTCTTCATCGA
C_ort_T2_90_125_a      ATGATTCACGAATATCTGCAATTCATATTACTTATCGCATTTCGCTGCGTTCTTCATCGA
                       ************************************************************

C_par_AY391843         TGCGAGAACCAAGAGATCCGTTGTTGAAAGTTTTGACTATTAAATAATC-GGTTGAC-AT
C_met_ATCC96143        TGCGAGAACCAAGAGATCCGTTGTTGAAAGTTTTGACTATTAGTTAATCAAGTTGACAAT
C_met_ATCC96144T       TGCGAGAACCAAGAGATCCGTTGTTGAAAGTTTTGACTATTAGTTAATCAAGTTGACAAT
C_met_Cp43             TGCGAGAACCAAGAGATCCGTTGTTGAAAGTTTTGACTATTAGTTAATCAAGTTGACAAT
C_ort_T1_MC0456_α      TGCGAGAACCAAGAGATCCGTTGTTGAAAGTTTTGACTATTAGTTAATC-AGTTGAC--T
C_ort_AY2_α            TGCGAGAACCAAGAGATCCGTTGTTGAAAGTTTTGACTATTAGTTAATC-AGTTGAC--T
C_ort_T1_Cp344_a       TGCGAGAACCAAGAGATCCGTTGTTGAAAGTTTTGACTATTAGTTAATC-AGTTGAC--T
C_ort_T1_Cp289_α       TGCGAGAACCAAGAGATCCGTTGTTGAAAGTTTTGACTATTAGTTAATC-AGTTGAC--T
C_ort_T1_Co288_a       TGCGAGAACCAAGAGATCCGTTGTTGAAAGTTTTGACTATTAGTTAATC-AGTTGAC--T
C_ort_T1_Cp85_α        TGCGAGAACCAAGAGATCCGTTGTTGAAAGTTTTGACTATTAGTTAATC-AGTTGAC--T
C_ort_T1_Cp47_a        TGCGAGAACCAAGAGATCCGTTGTTGAAAGTTTTGACTATTAGTTAATC-AGTTGAC--T
C_ort_T1_Cp25_a        TGCGAGAACCAAGAGATCCGTTGTTGAAAGTTTTGACTATTAGTTAATC-AGTTGAC--T
C_ort_T1_942211K_a     TGCGAGAACCAAGAGATCCGTTGTTGAAAGTTTTGACTATTAGTTAATC-AGTTGAC--T
C_ort_ATCC96139T       TGCGAGAACCAAGAGATCCGTTGTTGAAAGTTTTGACTATTAGTTAATC-AGTTGAC--T
C_ort_T2_Cp185_α       TGCGAGAACCAAGAGATCCGTTGTTGAAAGTTTTGACTATTAGTTAATC-AGTTGAC--T
C_ort_T2_Cp331_α       TGCGAGAACCAAGAGATCCGTTGTTGAAAGTTTTGACTATTAGTTAATC-AGTTGAC--T
C_ort_T2_Cp296_a       TGCGAGAACCAAGAGATCCGTTGTTGAAAGTTTTGACTATTAGTTAATC-AGTTGAC--T
C_ort_T2_Cp269_a       TGCGAGAACCAAGAGATCCGTTGTTGAAAGTTTTGACTATTAGTTAATC-AGTTGAC--T
C_ort_T2_Cp125_a/α     TGCGAGAACCAAGAGATCCGTTGTTGAAAGTTTTGACTATTAGTTAATC-AGTTGAC--T
C_ort_T2_90_125_a      TGCGAGAACCAAGAGATCCGTTGTTGAAAGTTTTGACTATTAGTTAATC-AGTTGAC--T
                       ******************************************  *****  ******  *
```

```
C_par_AY391843           TAAAT-AAAATTTGGTTGAGTTTAATCTCTGGCAGGCCCCATATAGAAGGCCTACCAAAG
C_met_ATCC96143          TAAATAAAAATTTGGTTGAGTTTAATCTCTGGCAGGCC-------GTGGGCCCACCAAAG
C_met_ATCC96144T         TAAATAAAAATTTGGTTGAGTTTAATCTCTGGCAGGCC-------GTGGGCCCACCAAAG
C_met_Cp43               TAAATAAAAATTTGGTTGAGTTTAATCTCTGGCAGGCC-------GTGGGCCCACCAAAG
C_ort_T1_MCO456_α        TAAAT-AAAATTTGGTTGAGTTTAATCTCTGGCAGGCC-------ATGGGCCCACCAAAG
C_ort_AY2_α              TAAAT-AAAATTTGGTTGAGTTTAATCTCTGGCAGGCC-------ATGGGCCCACCAAAG
C_ort_T1_Cp344_a         TAAAT-AAAATTTGGTTGAGTTTAATCTCTGGCAGGCC-------ATGGGCCCACCAAAG
C_ort_T1_Cp289_α         TAAAT-AAAATTTGGTTGAGTTTAATCTCTGGCAGGCC-------ATGGGCCCACCAAAG
C_ort_T1_Co288_a         TAAAT-AAAATTTGGTTGAGTTTAATCTCTGGCAGGCC-------ATGGGCCCACCAAAG
C_ort_T1_Cp85_α          TAAAT-AAAATTTGGTTGAGTTTAATCTCTGGCAGGCC-------ATGGGCCCACCAAAG
C_ort_T1_Cp47_a          TAAAT-AAAATTTGGTTGAGTTTAATCTCTGGCAGGCC-------ATGGGCCCACCAAAG
C_ort_T1_Cp25_a          TAAAT-AAAATTTGGTTGAGTTTAATCTCTGGCAGGCC-------ATGGGCCCACCAAAG
C_ort_T1_942211K_a       TAAAT-AAAATTTGGTTGAGTTTAATCTCTGGCAGGCC-------ATGGGCCCACCAAAG
C_ort_ATCC96139T         TAAAT-AAAATTTGGTTGAGTTTAATCTCTGGCAGGCC-------ATGGGCCCACCAAAG
C_ort_T2_Cp185_α         TAAATAAAAATTTGGTTGAGTTTAATCTCTGGCAGGCC-------ATGGGCCCACCAAAG
C_ort_T2_Cp331_α         TAAATAAAAATTTGGTTGAGTTTAATCTCTGGCAGGCC-------ATGGGCCCACCAAAG
C_ort_T2_Cp296_a         TAAATAAAAATTTGGTTGAGTTTAATCTCTGGCAGGCC-------ATGGGCCCACCAAAG
C_ort_T2_Cp269_a         TAAATAAAAATTTGGTTGAGTTTAATCTCTGGCAGGCC-------ATGGGCCCACCAAAG
C_ort_T2_Cp125_a/α       TAAATAAAAATTTGGTTGAGTTTAATCTCTGGCAGGCC-------ATGGGCCCACCAAAG
C_ort_T2_90_125_a        TAAATAAAAATTTGGTTGAGTTTAATCTCTGGCAGGCC-------ATGGGCCCACCAAAG
                         ***** ********************************          **** *******

C_par_AY391843           CAAAGTTTTC--AAAAAAAGAAAAACACATGTGTAAGAAAAAA-----TGCAGTTAAGCA
C_met_ATCC96143          CAAAGTTTTC-AAAAAAAAGAAAAACACATGTGTAGAAAGAAAAGGATTGCAGTTAAGCA
C_met_ATCC96144T         CAAAGTTTTC-AAAAAAAAGAAAAACACATGTGTAGAAAGAAAAGGATTGCAGTTAAGCA
C_met_Cp43               CAAAGTTTTC-AAAAAAAAGAAAAACACATGTGTAGAAAGAAAAGGATTGCAGTTAAGCA
C_ort_T1_MCO456_α        CAAAGTTTTCAAAAAAAAAGAAAAACACATGTGTAAAAAAA-------TGCAGTTAAGCA
C_ort_AY2_α              CAAAGTTTTCAAAAAAAAAGAAAAACACATGTGTAAAAAAA-------TGCAGTTAAGCA
C_ort_T1_Cp344_a         CAAAGTTTTCAAAAAAAAAGAAAAACACATGTGTAAAAAAA-------TGCAGTTAAGCA
C_ort_T1_Cp289_α         CAAAGTTTTCAAAAAAAAAGAAAAACACATGTGTAAAAAAA-------TGCAGTTAAGCA
C_ort_T1_Co288_a         CAAAGTTTTCAAAAAAAAAGAAAAACACATGTGTAAAAAAA-------TGCAGTTAAGCA
C_ort_T1_Cp85_α          CAAAGTTTTCAAAAAAAAAGAAAAACACATGTGTAAAAAAA-------TGCAGTTAAGCA
C_ort_T1_Cp47_a          CAAAGTTTTCAAAAAAAAAGAAAAACACATGTGTAAAAAAA-------TGCAGTTAAGCA
C_ort_T1_Cp25_a          CAAAGTTTTCAAAAAAAAAGAAAAACACATGTGTAAAAAAA-------TGCAGTTAAGCA
C_ort_T1_942211K_a       CAAAGTTTTCAAAAAAAAAGAAAAACACATGTGTAAAAAAA-------TGCAGTTAAGCA
C_ort_ATCC96139T         CAAAGTTTTCAAAAAAAAAGAAAAACACATGTGTAAAAAAA-------TGCAGTTAAGCA
C_ort_T2_Cp185_α         CAAAGTTTTCAAAAAAAAAGAAAAACACATGTGTAAAAAAAA------TGCAGTTAAGCA
C_ort_T2_Cp331_α         CAAAGTTTTCAAAAAAAAAGAAAAACACATGTGTAAAAAAAA------TGCAGTTAAGCA
C_ort_T2_Cp296_a         CAAAGTTTTCAAAAAAAAAGAAAAACACATGTGTAAAAAAAA------TGCAGTTAAGCA
C_ort_T2_Cp269_a         CAAAGTTTTCAAAAAAAAAGAAAAACACATGTGTAAAAAAAA------TGCAGTTAAGCA
C_ort_T2_Cp125_a/α       CAAAGTTTTCAAAAAAAAAGAAAAACACATGTGTAAAAAAAA------TGCAGTTAAGCA
C_ort_T2_90_125_a        CAAAGTTTTCAAAAAAAAAGAAAAACACATGTGTAAAAAAA-------TGCAGTTAAGCA
                         **********  ***********************  ** *       ************

C_par_AY391843           CTTTTC
C_met_ATCC96143          ACCTTC
C_met_ATCC96144T         ACCTTC
C_met_Cp43               ACCTTC
C_ort_T1_MCO456_α        -CTTTC
C_ort_AY2_α              -CTTTC
C_ort_T1_Cp344_a         -CTTTC
C_ort_T1_Cp289_α         -CTTTC
C_ort_T1_Co288_a         -CTTTC
C_ort_T1_Cp85_α          -CTTTC
C_ort_T1_Cp47_a          -CTTTC
C_ort_T1_Cp25_a          -CTTTC
C_ort_T1_942211K_a       -CTTTC
C_ort_ATCC96139T         -CTTTC
C_ort_T2_Cp185_α         -CTTTC
C_ort_T2_Cp331_α         -CTTTC
C_ort_T2_Cp296_a         -CTTTC
C_ort_T2_Cp269_a         -CTTTC
C_ort_T2_Cp125_a/α       -CTTTC
C_ort_T2_90_125_a        -CTTTC
                            ***
```

Supplementary figure S3: Mating type locus in *C. orthopsilosis*

The MTL sequences were obtained from GenBank (HQ696678 – HQ696682).

A. Structural analysis of MTL locus in *C. orthopsilosis*

We have aligned MTL from six *C. orthopsilosis* strains. MTL from *C. metapsilosis* ATCC 96143 have been included as outgroup. Synteny within MTL is maintained across species. Strains were assigned as MTLa or MTLα by structural analysis of MTL locus. In MTLa synteny block colored blue precedes the purple one. In contrast, in MTLα, blue block occurs after the purple one. Therefore, both *C. orthopsilosis* strains under inversigation, MCO456 and AY2, are MTLα. The alignment was reconstructed using MAUVE v2.3.1 (Angiuoli and Salzberg, 2011).

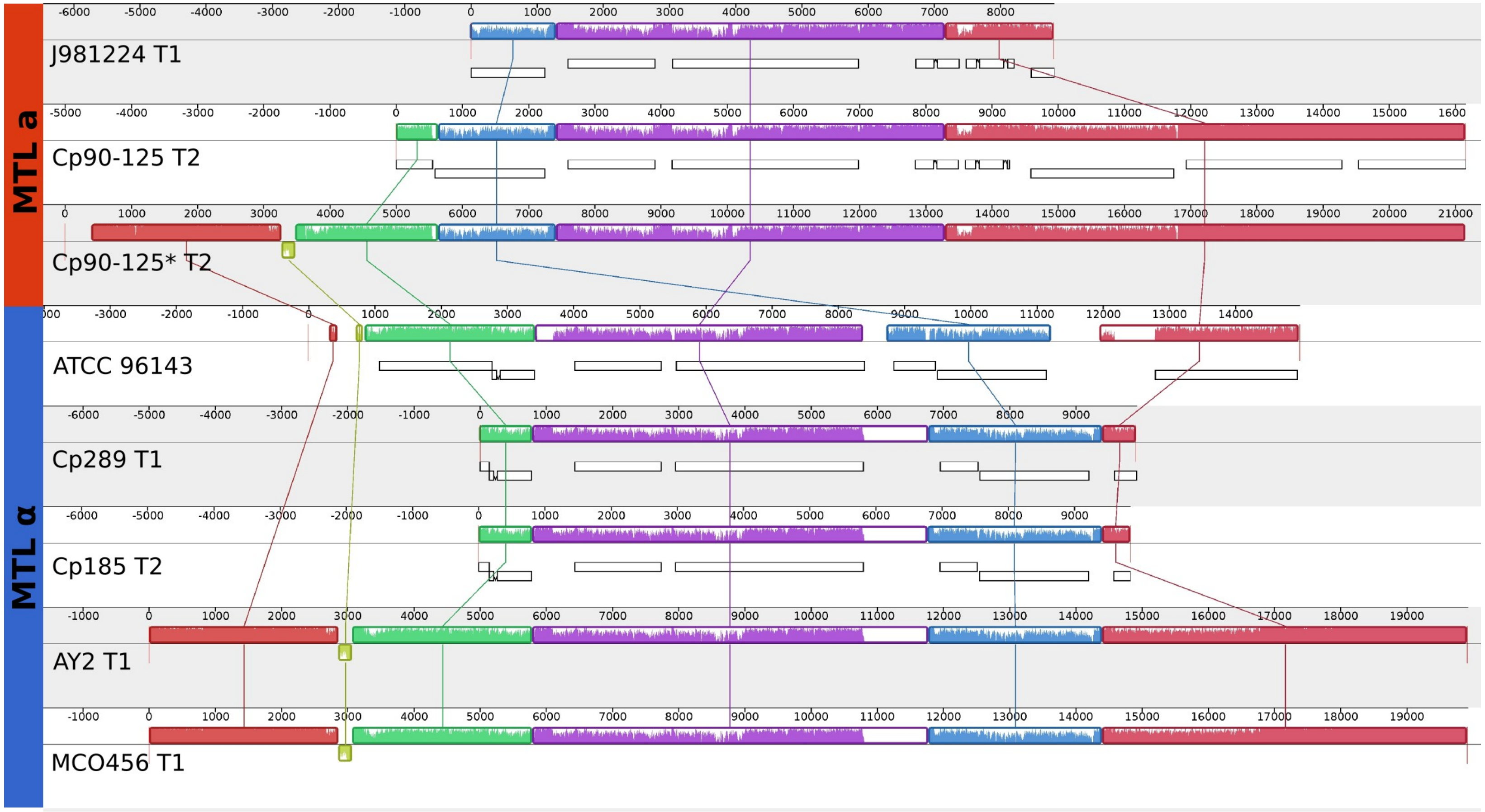

B. Type 1 / Type 2 assignment

MTLα of Type 1 and Type 2 are 4.66% diverged. Interestingly, MCO456 and AY2 are 100% identical in sequence to Cp289 (MTLα; Type 1) and therefore should be considered Type 1. The multiple sequence of MTL was reconstructed using Muscle v3.8.31 (Edgar, 2004). Alignment was trimmed using Trimal in version 1.4 to remove poorly aligned columns (Capella-Gutiérrez et al., 2009). Trimmed alignment consited of 9,573 columns. Maximum Likelihood phylogenetic tree was reconstructed using RAxML 7.2.8 using GTRCAT model (Stamatakis et al., 2005).

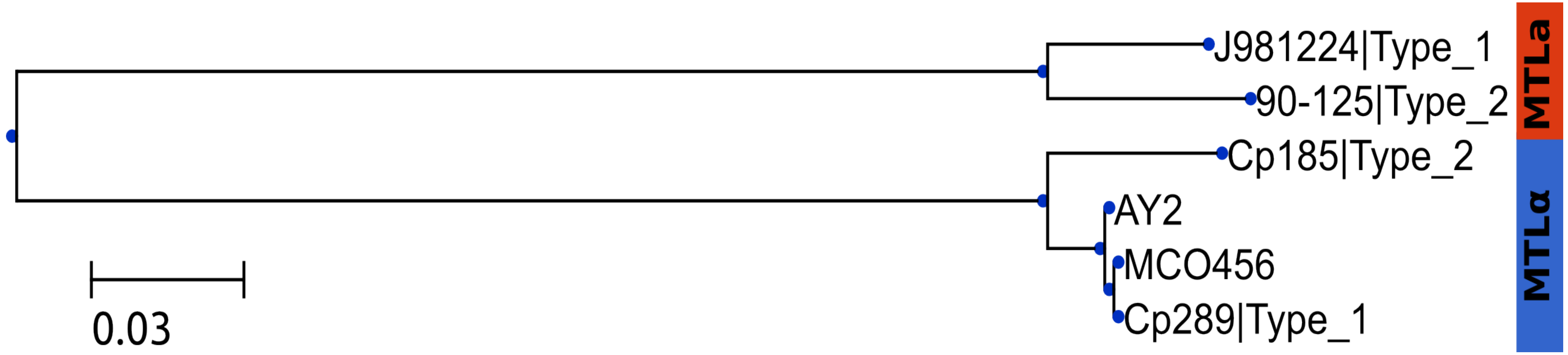

Supplementary figure S4: The largest identified duplication (DUP2)

Features of the largest chromosome (HE681719) are plotted. Two upper panels present: i) copy number as log2 of observed vs expected depth of coverage in 1kb windows (blue line) and ii) haplotype assignment (white – haplotype A/Type 2; grey – haplotype B/Type 1, orange – heterozygous) for two *C. orthopsilosis* strains, 90-125 and MCO456, respectively. Bottom track present coding genes (grey bars) for +/- strand and GC-content in 1kb windows (blue plot). The largest duplication (DUP2) of 238kb can be easily spotted in strain 90-125.

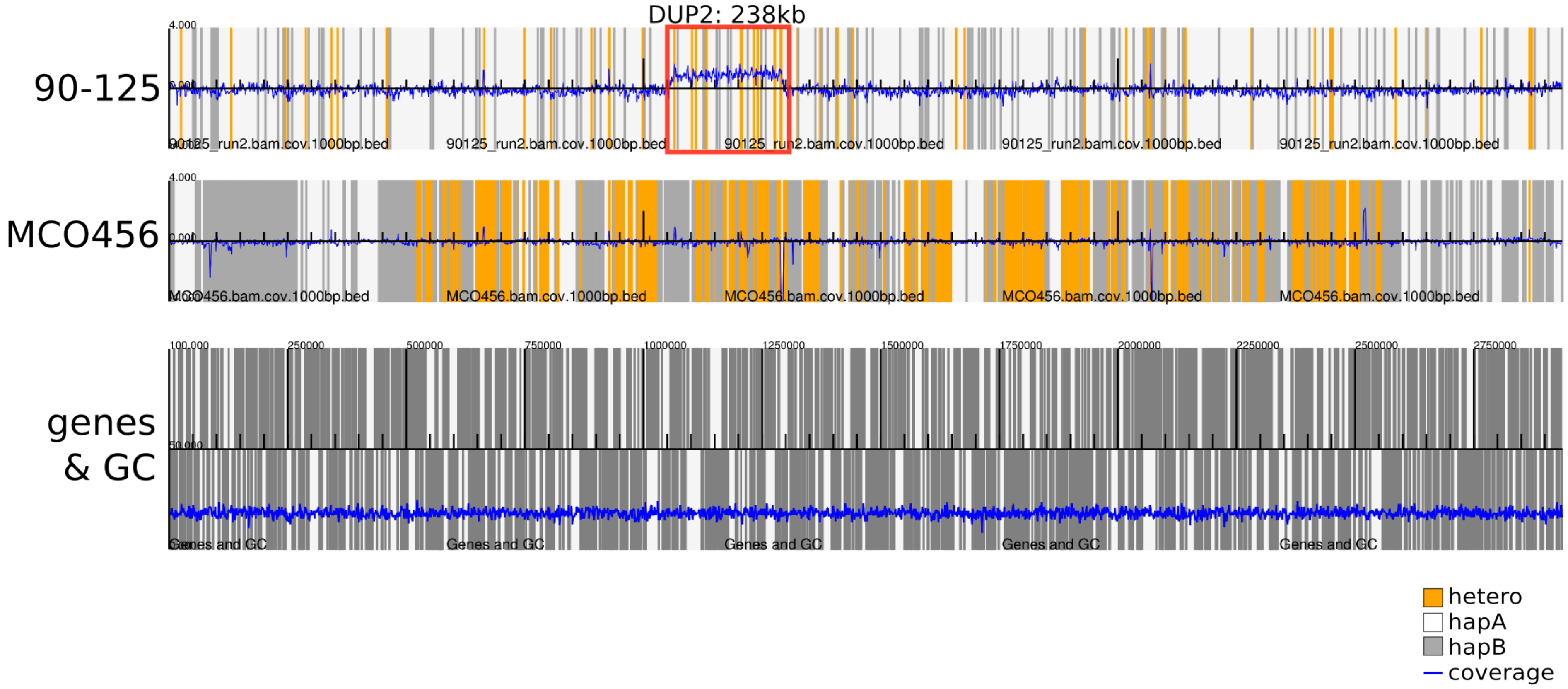

Supplementary figure S5: Ploidy analyses

Distributions of frequencies of read counts at biallelic SNPs in *C. orthopsilosis* MCO456 were plotted. We observed single peak around 50% for each chromosome, indicating MCO456 is diploid.

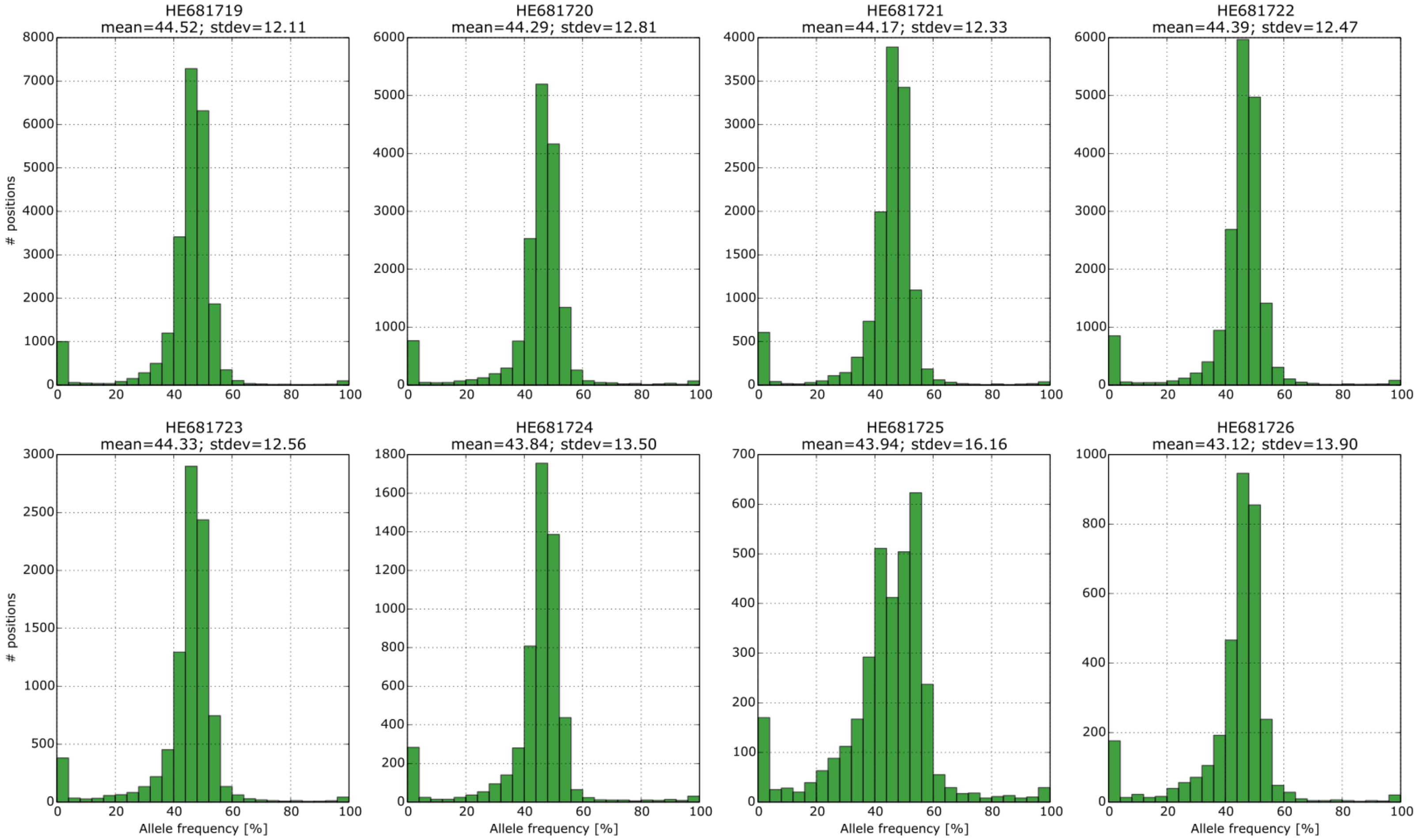

Supplementary figure S6: *C. orthopsilosis* MCO456 chromosomes characteristics

Relationships between size of C. orthopsilosis MCO456 chromosomes and: i) fraction of chromosome being heterozygous (blue; top panel) and ii) mean length of LOH tracks (red, bottom panel). Mean length of LOH tracks is negatively correlated to chromosome length (Spearman r=-0.76, p<0.028), and level of heterozygosity is positively correlated to chromosome length (Spearman r=0.71, p<0.0465).

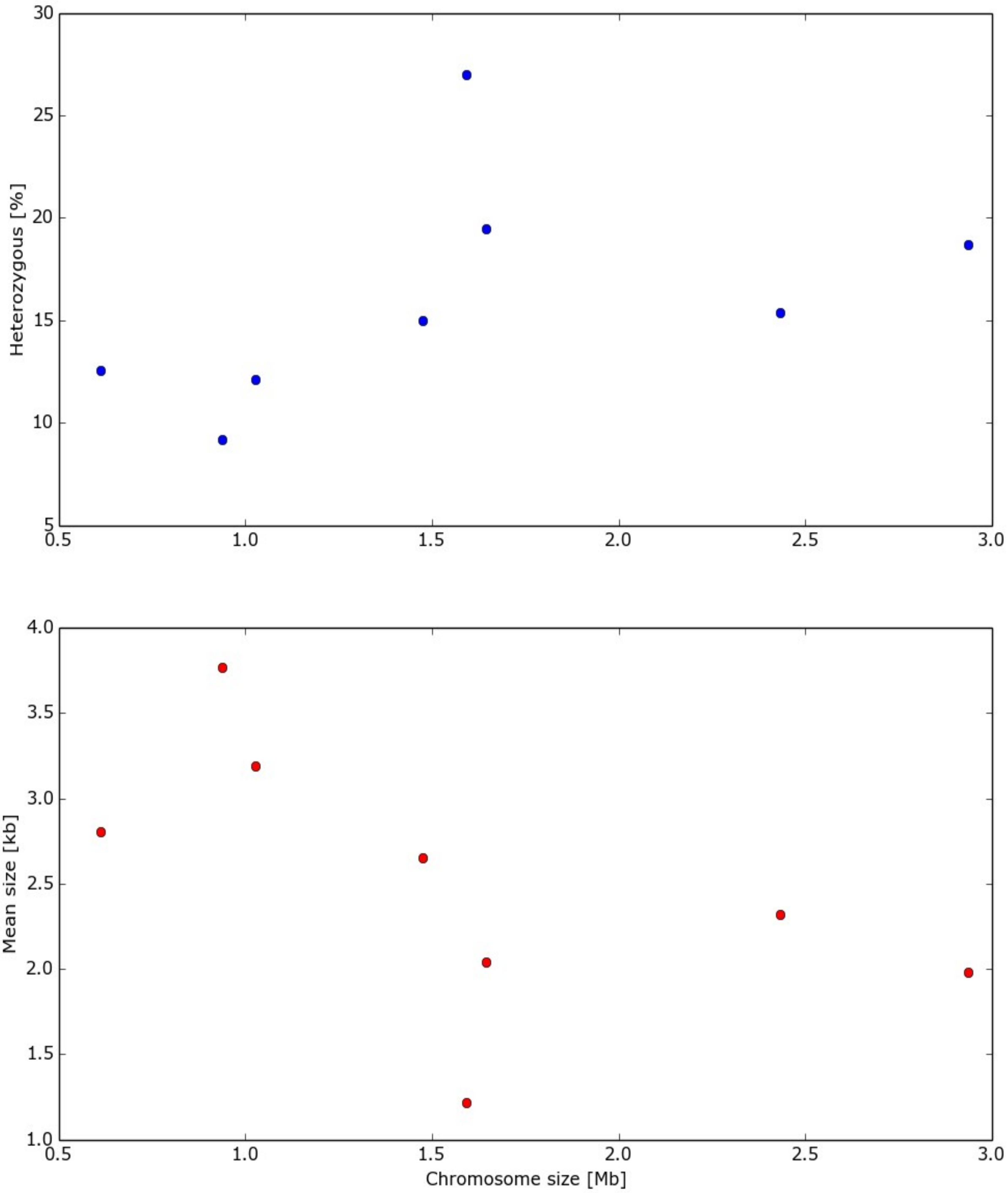

References

Angiuoli, S.V., Salzberg, S.L., 2011. Mugsy: fast multiple alignment of closely related whole genomes. Bioinforma. Oxf. Engl. 27, 334–342.

Capella-Gutiérrez, S., Silla-Martínez, J.M., Gabaldón, T., 2009. trimAl: a tool for automated alignment trimming in large-scale phylogenetic analyses. Bioinforma. Oxf. Engl. 25, 1972–1973.

Edgar, R.C., 2004. MUSCLE: multiple sequence alignment with high accuracy and high throughput. Nucleic Acids Res. 32, 1792–1797.

Sai, S., Holland, L.M., McGee, C.F., Lynch, D.B., Butler, G., 2011. Evolution of mating within the Candida parapsilosis species group. Eukaryot. Cell 10, 578–587.

Stamatakis, A., Ludwig, T., Meier, H., 2005. RAxML-III: a fast program for maximum likelihood-based inference of large phylogenetic trees. Bioinforma. Oxf. Engl. 21, 456–463.
