# Supplementary material for: Genome Comparison of Candida orthopsilosis Clinical Strains Reveals the Existence of Hybrids between Two Distinct Subspecies
Source: Genome Biol Evol. 2014 Apr 18;6(5):1069–78. doi: 10.1093/gbe/evu082 (PMC4040990; doi:10.1093/gbe/evu082)
Supplement: Supplementary Data [file supp_evu082_SuppFile3.pdf]

Supplementary file 3

Candida orthopsilosis chromosomes graphs

For each chromosome we have plotted: i) coding genes for +/- strand (grey bars) and GC-content in 1kb windows (blue plot) in the bottom track and ii) log2 of observed vs expected value in 1kb windows for: depth of coverage (blue), homozygous SNPs (grey) and heterozygous SNPs (orange) in the four top tracks. Two replicas were analysed for both strains: MCO456 (methodological replicas aligned with bowtie2 and bwa) and 90-125 (two independent sequencing runs).

Examples of large duplications and deletions have been annotated ie. rDNA cluster (DUP29) in both strains, DUP2 in 90-125 (HE681719:1,054,593-1,293,449) or DEL51 in MCO456 (HE681721:597,463-604,763).

Examples of haplotype A, haplotype B and heterozygous regions in HE681719 and HE681725 from MCO456 have been annotated. MCO456 genome is a mixture of heterozygous (orange plot above 0), haplotype B (grey plot above 0) and haplotype A (both, grey and orange plots below 0) regions. Interestingly, left arm of chromosome HE681725 starts from 130kb that is haplotype B, followed by nearly 200kb of haplotype A, where rDNA cluster (DUP29) is found. rDNA is therefore homozygous and haplotype A in MCO456, so unlike the most of the chromosome HE681725 it originates from Type 2 parental, similar to *C. orthopsilosis* 90-125.

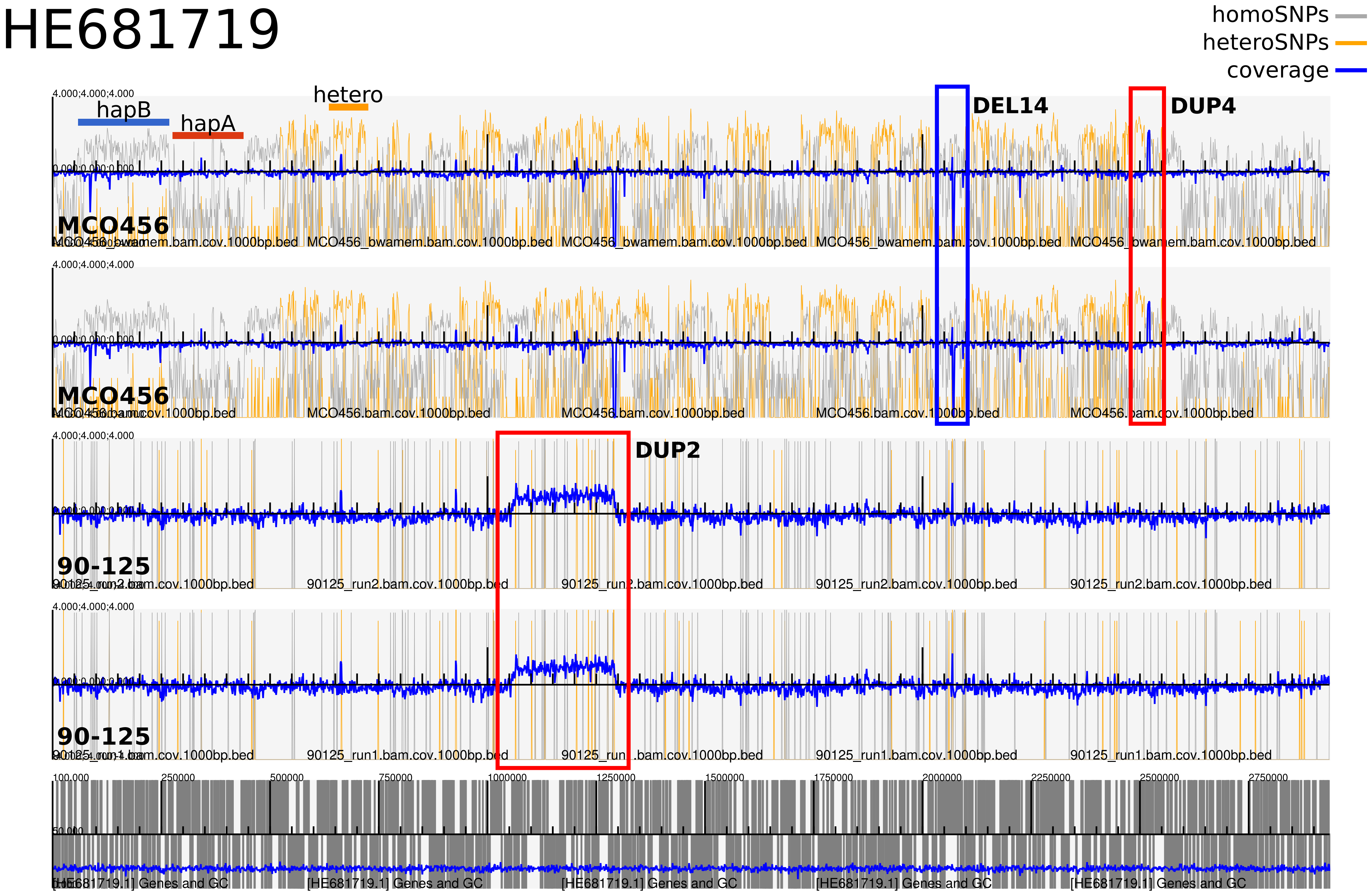

HE681719
homoSNPs
heteroSNPs
coverage
hapB
hapA
hetero
DEL14
DUP4
MCO456
MCO456_bwamem.bam.cov.1000bp.bed
MCO456.bam.cov.1000bp.bed
DUP2
90-125
90125_run2.bam.cov.1000bp.bed
90125_run1.bam.cov.1000bp.bed
[HE681719.1] Genes and GC

HE681720

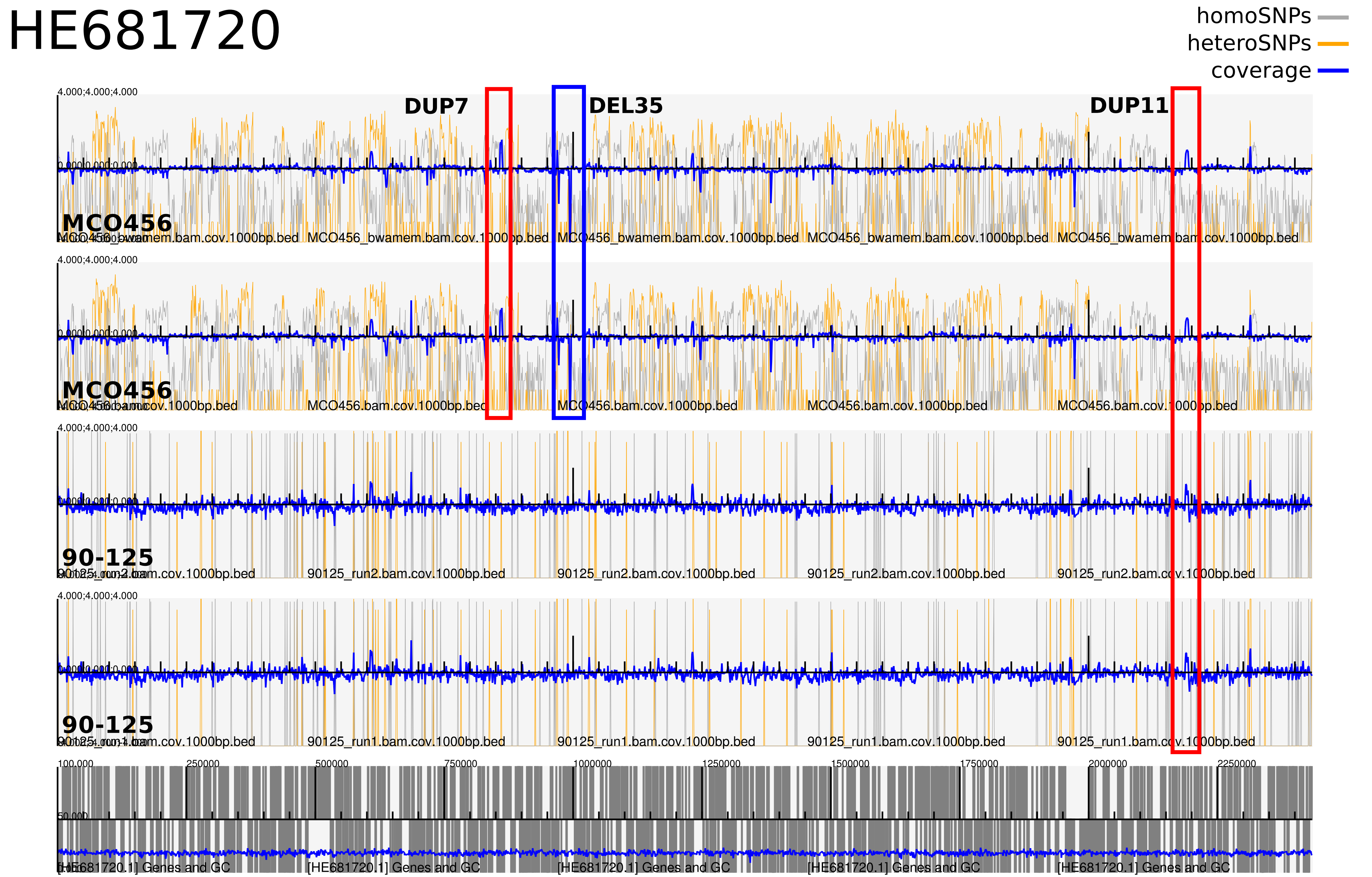
homoSNPs
heteroSNPs
coverage
DUP7
DEL35
DUP11
MCO456
MCO456_bwamem.bam.cov.1000bp.bed
MCO456
MCO456.bam.cov.1000bp.bed
90-125
90125_run2.bam.cov.1000bp.bed
90-125
90125_run1.bam.cov.1000bp.bed
250000
500000
750000
1000000
1250000
1500000
1750000
2000000
2250000
[HE681720.1] Genes and GC

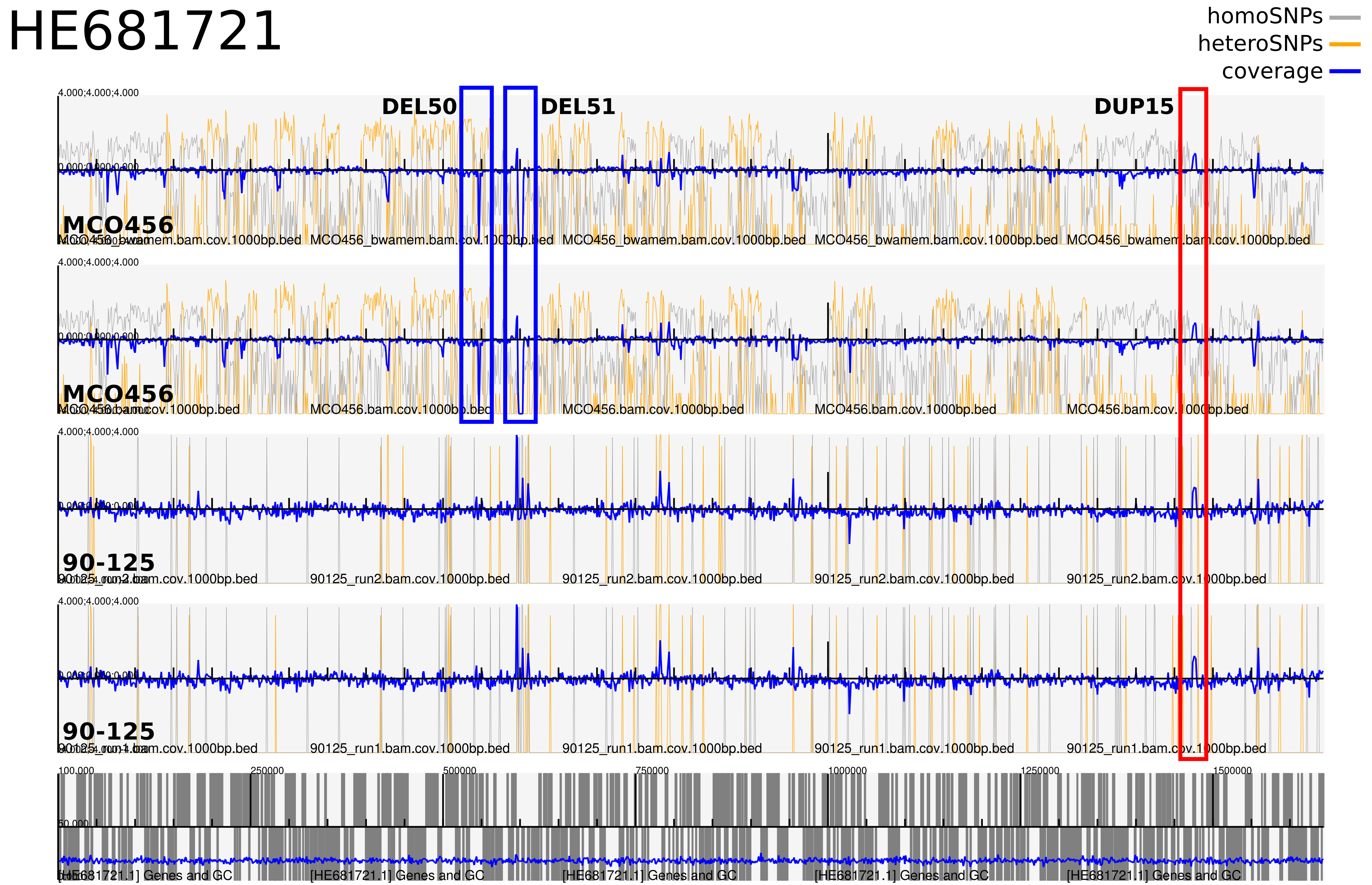
HE681721
homoSNPs
heteroSNPs
coverage
DEL50
DEL51
DUP15
MCO456
MCO456_bwamem.bam.cov.1000bp.bed
MCO456
MCO456.bam.cov.1000bp.bed
90-125
90125_run2.bam.cov.1000bp.bed
90-125
90125_run1.bam.cov.1000bp.bed
250000
500000
750000
1000000
1250000
1500000
[HE681721.1] Genes and GC

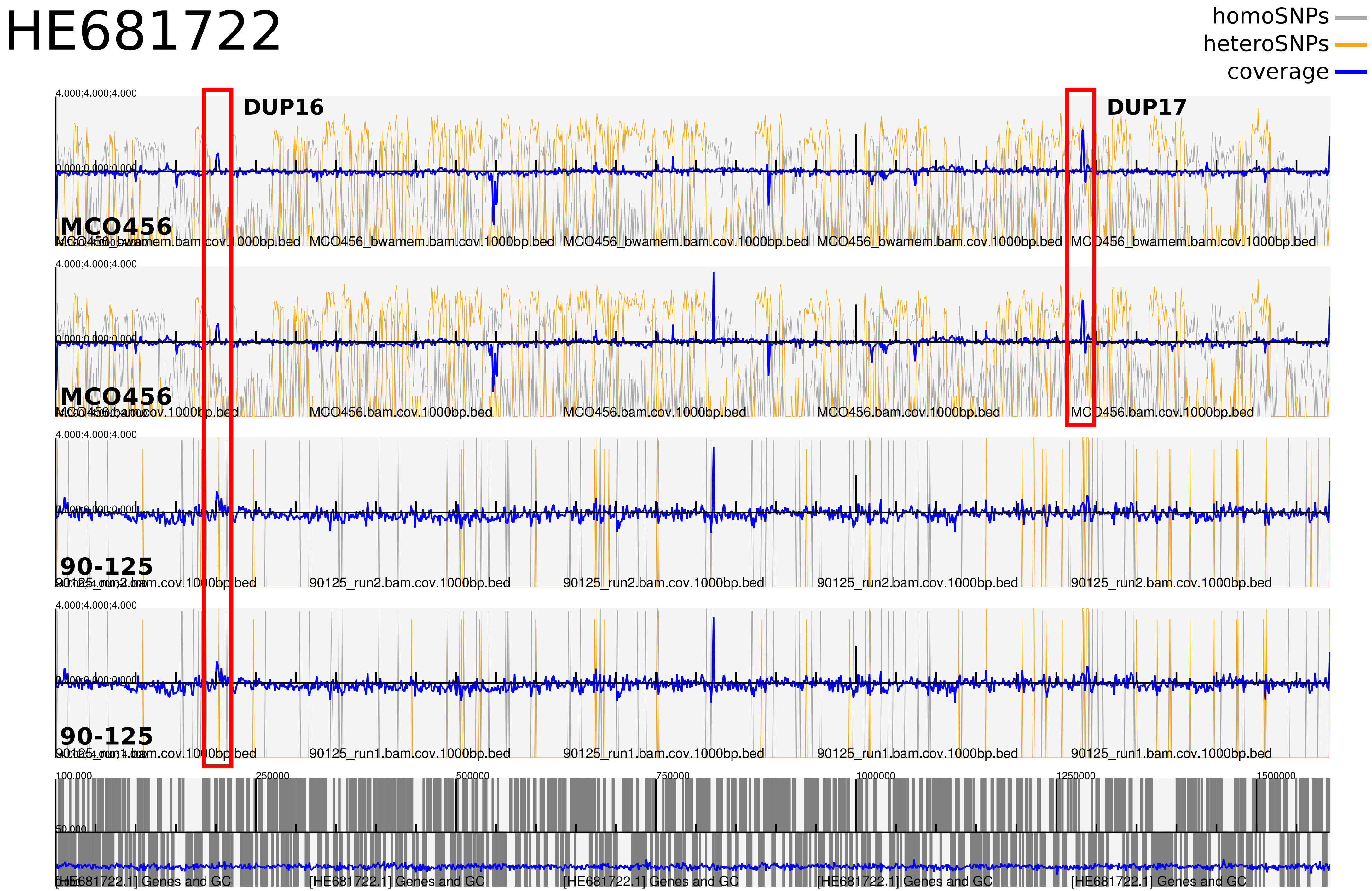
HE681722
homoSNPs
heteroSNPs
coverage
DUP16
DUP17
4.000;4.000;4.000
0.000;0.000;0.000
MCO456
MCO456_bwamem.bam.cov.1000bp.bed
MCO456
MCO456.bam.cov.1000bp.bed
90-125
90125_run2.bam.cov.1000bp.bed
90-125
90125_run1.bam.cov.1000bp.bed
100.000
50.000
250000
500000
750000
1000000
1250000
1500000
[HE681722.1] Genes and GC

HE681723

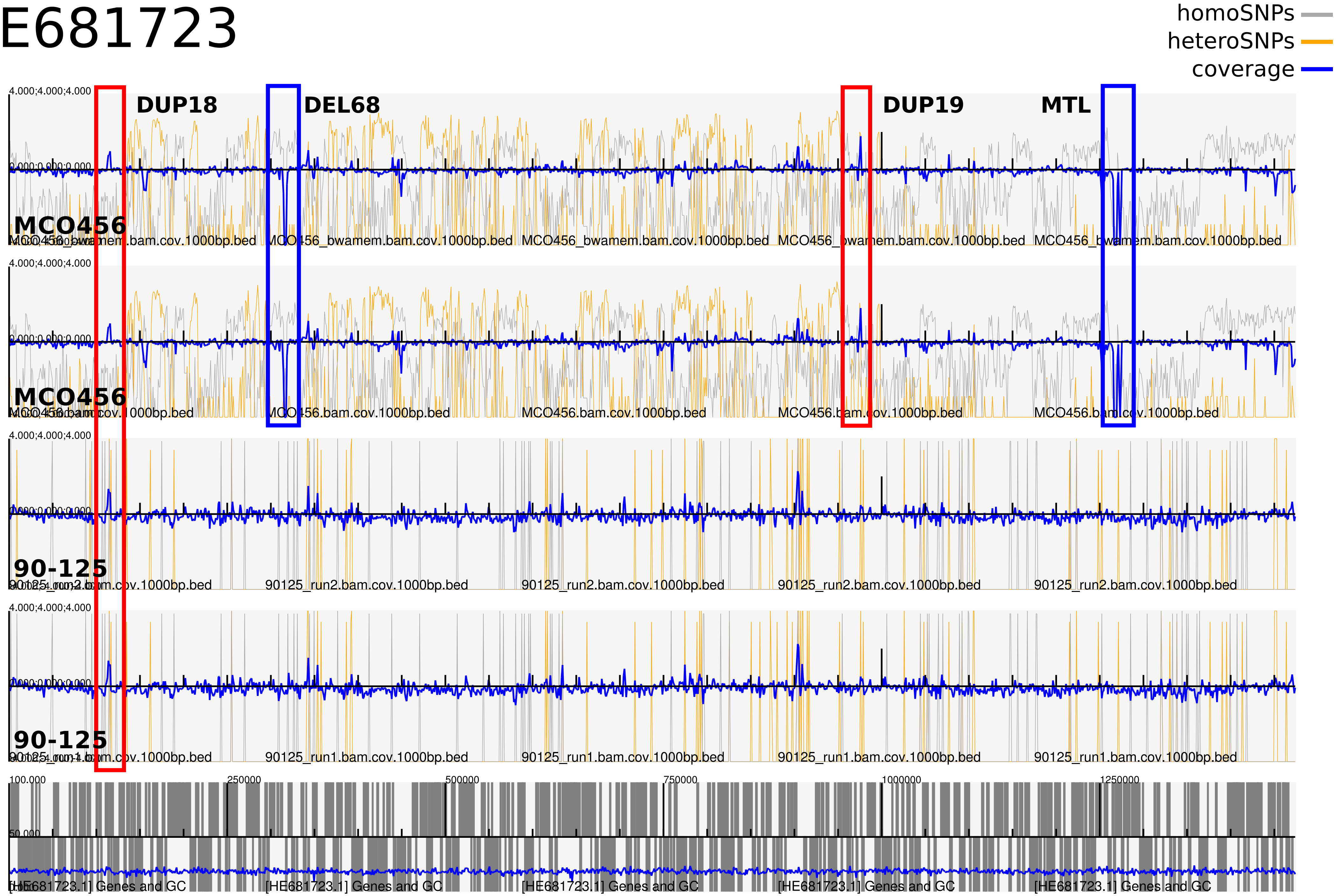
homoSNPs
heteroSNPs
coverage
DUP18
DEL68
DUP19
MTL
MCO456
MCO456_bwamem.bam.cov.1000bp.bed
MCO456
MCO456.bam.cov.1000bp.bed
90-125
90125_run2.bam.cov.1000bp.bed
90-125
90125_run1.bam.cov.1000bp.bed
4.000;4.000;4.000
0.000;0.000;0.000
100.000
50.000
250000
500000
750000
1000000
1250000
[HE681723.1] Genes and GC

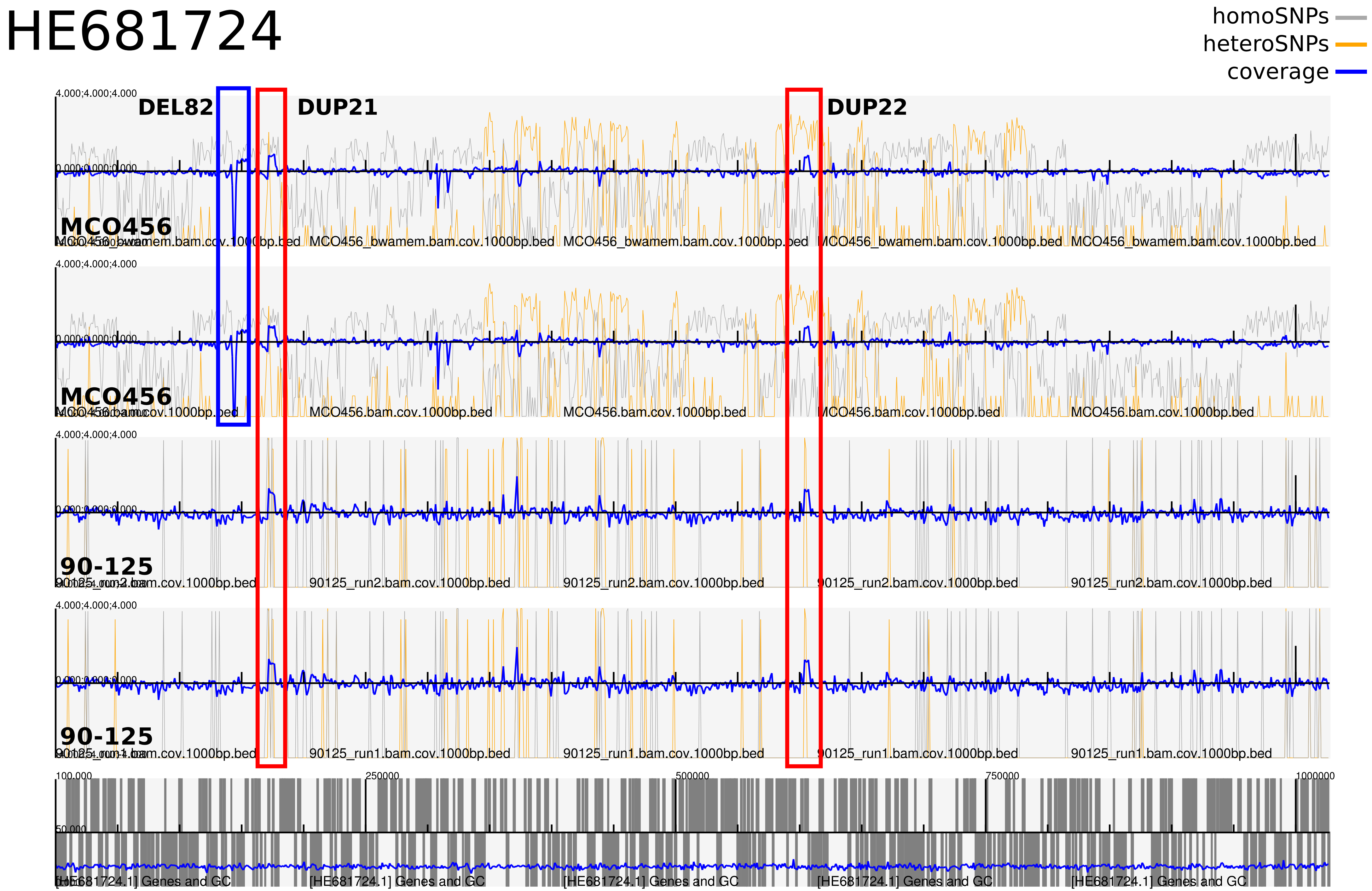

HE681724
homoSNPs
heteroSNPs
coverage
DEL82
DUP21
DUP22
MCO456
MCO456_bwamem.bam.cov.1000bp.bed
MCO456
MCO456.bam.cov.1000bp.bed
90-125
90125_run2.bam.cov.1000bp.bed
90-125
90125_run1.bam.cov.1000bp.bed
4.000;4.000;4.000
0.000;0.000;0.000
100.000
50.000
250000
500000
750000
1000000
[HE681724.1] Genes and GC

HE681725

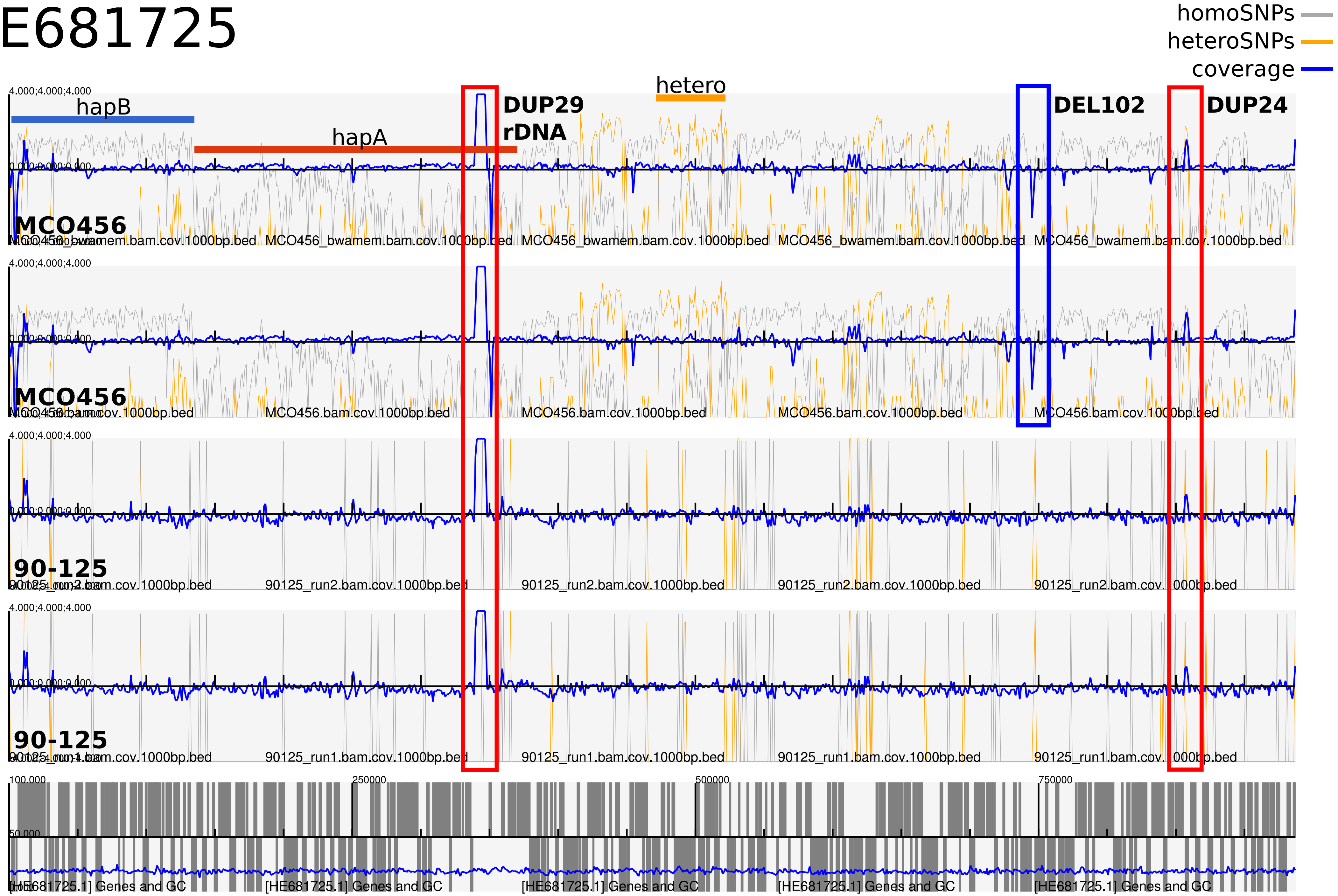
homoSNPs
heteroSNPs
coverage
hapB
hapA
hetero
DUP29
rDNA
DEL102
DUP24
MCO456
MCO456_bwamem.bam.cov.1000bp.bed
MCO456
MCO456.bam.cov.1000bp.bed
90-125
90125_run2.bam.cov.1000bp.bed
90-125
90125_run1.bam.cov.1000bp.bed
100.000
250000
500000
750000
50.000
[HE681725.1] Genes and GC

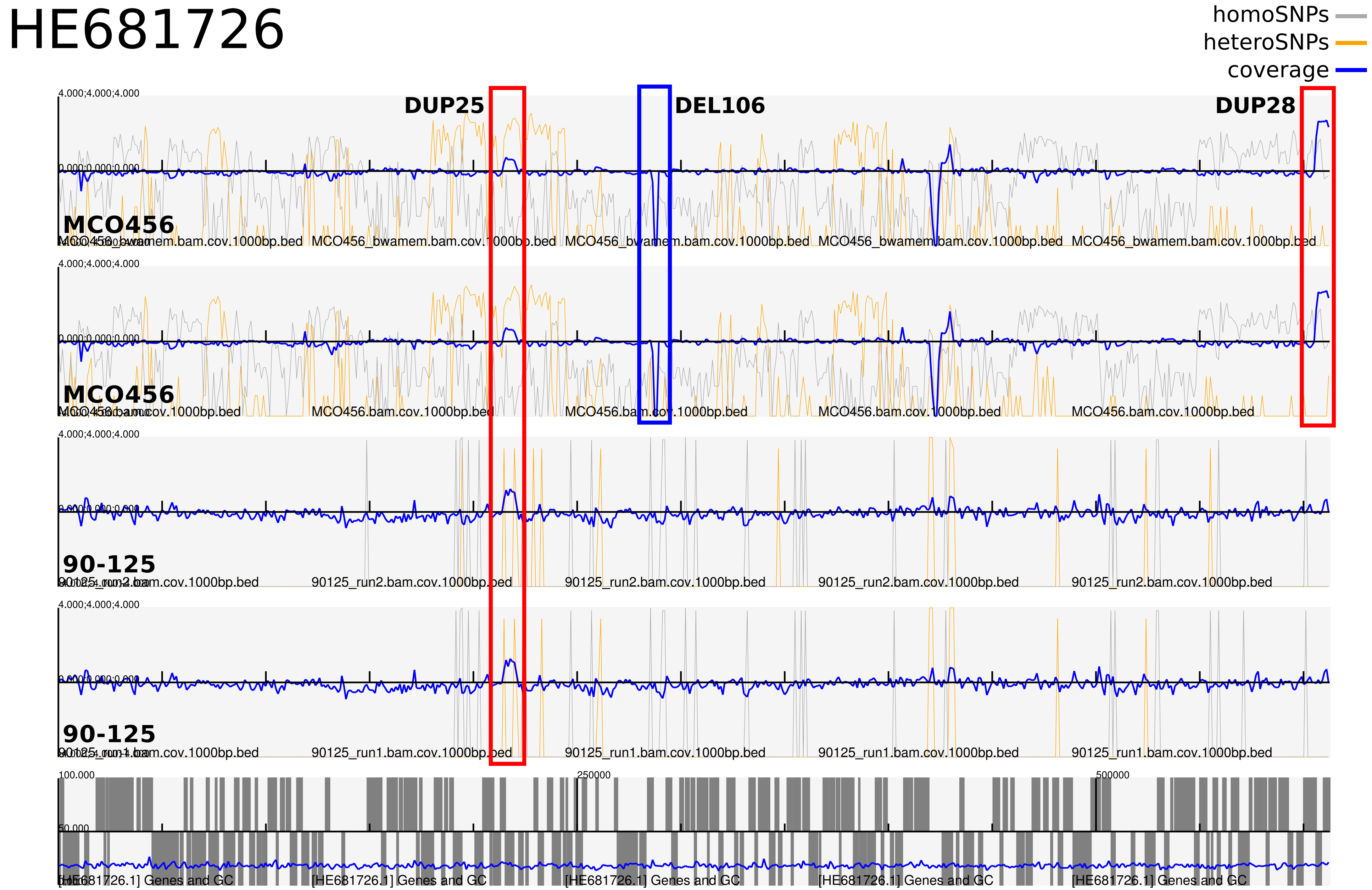

HE681726
homoSNPs
heteroSNPs
coverage
DUP25
DEL106
DUP28
MCO456
MCO456_bwamem.bam.cov.1000bp.bed
MCO456.bam.cov.1000bp.bed
90-125
90125_run2.bam.cov.1000bp.bed
90125_run1.bam.cov.1000bp.bed
[HE681726.1] Genes and GC
4.000;4.000;4.000
0.000;0.000;0.000
100.000
50.000
250000
500000
