# Supplementary material for: Genome Comparison of Candida orthopsilosis Clinical Strains Reveals the Existence of Hybrids between Two Distinct Subspecies
Source: Genome Biol Evol. 2014 Apr 18;6(5):1069–78. doi: 10.1093/gbe/evu082 (PMC4040990; doi:10.1093/gbe/evu082)
Supplement: Supplementary Data [file supp_evu082_SuppFile4.pdf]

Supplementary file 4

Recombinations between two haplotypes in *C. orthopsilosis* MCO456 and AY2

We have scanned alignments of heterozygous regions (two haplotypes) from MCO456 and AY2 with 90-125 chromosomes (CANOR) in order to identify 100 bases windows in which haplotype B has higher identity to 90-125 than haplotype A. These are putative recombination sites between two haplotypes. We have found 261 such regions in 157 alignments. Subsequently, we have realigned such regions by Muscle (http://www.ebi.ac.uk/Tools/msa/muscle/; Edgar, 2004). Conserved sites are in cyan. Columns informing about recombinations are marked with caret (^) symbol beneath.

Interestingly, most of the observed SNP patterns are shared by both, MCO456 and AY2, therefore it is more parsimonius to assume that the recombination took place in the hybrid ancestor of the two. Note, some of the observed patterns, especially these confirmed by single SNP, could be explained by gaining SNP in haplotype A in conserved region between both haplotypes and 90-125. But the cases involving more SNPs and INDELs (ie. HE681719 [1 event, 2 SNPs + INDEL] or HE681722 [1 event, 1 SNP + INDEL]) would be difficult to explain by such scenario.

HE681719 [1 event, 2 SNPs + INDEL]....2
HE681723 [2 events, 1 SNP each]....3
HE681725 [3 events; 1+ SNP each]....5
HE681722 [1 event, 1 SNP + INDEL]....7
HE681719 [1 event, 2 SNPs]....7
HE681721 [5 events, 7 SNPs]....8
HE681721 [1 event, 1 SNP + LOH....9

HE681719 [1 event, 2 SNPs + INDEL]

```
CANOR.HE681719                CAGCTGGGCCAGTGAGTGGTTGCTGATGTGGCTTATGTTGATGAGACTGCACCACGGACA
AY2_hapA.contig00554          CAGCTGGGCCAGTGAGTGGTTGCTGATGTGGCTTATGTTGATGAGACTGCACCACGGACA
MC0456_hapB.contig00994       CAGCTGGGCCAGTGAGTGGTTGCTGATGTGGCTTATGTTGATGAGACTGCACCACGGACA
MC0456_hapA.contig00792       CAGCTGGGCCAGTtAGTGGTTGCTGATGTGGCTTATGTTGATGAGACTGCACCACaGACA
AY2_hapB.contig00598          CAGCTGGGCCAGTtAGTGGTTGCTGATGTGGCTTATGTTGATGAGACTGCACCACaGACA

CANOR.HE681719                CATTTGGGTGTTGTGATTCCAGTGGACTCTTTTTCGATGTAGGAGAGTAATGCTTGACAC
AY2_hapA.contig00554          CATTTGGGTGTTGTGATTCCAGTGGACTCTTTTTCGATGTAGGAGAGTAATGCTTGACAC
MC0456_hapB.contig00994       CATTTGGGTGTTGTGATTCCAGTGGACTCTTTTTCGATGTAGGAGAGTAATGCTTGACAC
MC0456_hapA.contig00792       CATTTGGaTGTTGTGATTCCAGaGGACTCTTTTTtGATGTAGGAGAGTAATGCTTGACAC
AY2_hapB.contig00598          CATTTGGaTGTTGTGATTCCAGaGGACTCTTTTTtGATGTAGGAGAGTAATGCTTGACAC

CANOR.HE681719                CTTCATCACTCATGTTTTCCTTATCCAGTGAAAGACTAGTACTACTTTGTTGAATCTTCA
AY2_hapA.contig00554          CTTCATCACTCATGTTTTCCTTATCCAGTGAAAGACTAGTACTACTTTGTTGAATCTTCA
MC0456_hapB.contig00994       CTTCATCACTCATGTTTTCCTTATCCAGTGAAAGACTAGTACTACTTTGTTGAATCTTCA
MC0456_hapA.contig00792       CTTCATCACTCATGTTTTCCTTATCCAGTGAAAGgCTAGTACTACTTTGTTGAATCTTCA
AY2_hapB.contig00598          CTTCATCACTCATGTTTTCCTTATCCAGTGAAAGgCTAGTACTACTTTGTTGAATCTTCA

CANOR.HE681719                AATCGGGATGAACTTTGTATGTTGGTGTTCTGTTGAAACTAGCACCACCGGGTAATGAAG
AY2_hapA.contig00554          AATCGGGATGAACTTTGTATGTTGGTGTTCTGTTGAAACTAGCACCACCGGGTAATGAAG
MC0456_hapB.contig00994       AATCGGGATGAACTTTGTATGTTGGTGTTCTGTTGAAACTAGCACCACCGGGTAATGAAG
MC0456_hapA.contig00792       AATCtGGATGAACTTTGTATGTTGGTGTTCTGTTaAAACTAGCACCACCaGGTAATGAAG
AY2_hapB.contig00598          AATCtGGATGAACTTTGTATGTTGGTGTTCTGTTaAAACTAGCACCACCaGGTAATGAAG

CANOR.HE681719                GTGGCACTTGTTGCGGTTTAGATGTAGATTGTTGACGTTGATGACTACGCAATGGTTGTT
AY2_hapA.contig00554          GTGGCACTTGTTGCGGTTTAGATGTAGATTGTTGACGTTGATGACTACGCAATGGTTGTT
MC0456_hapB.contig00994       GTGGCACTTGTTGCGGTTTAGATGTAGATTGTTGACGTTGATGACTACGCAATGGTTGTT
MC0456_hapA.contig00792       GTGGCACTTGTTGaGGTTTAGATGTAGATTGTTGACGTTGATGACTACGCAATGGTTGTT
AY2_hapB.contig00598          GTGGCACTTGTTGaGGTTTAGATGTAGATTGTTGACGTTGATGACTACGCAATGGTTGTT

CANOR.HE681719                GT------TGTTGTTGCTGCTGCTGCTGCTGCTGCTTGGATATACCTCCACTTGATCCTG
AY2_hapA.contig00554          GTTGCTGCTGcTGcTGCTGCTGCTGCTGCTGCTGCTTGGATATACCTCCACTTGATCCTG
MC0456_hapB.contig00994       GTTGCTGCTGcTGcTGCTGCTGCTGCTGCTGCTGCTTGGATATACCTCCACTTGATCCTG
MC0456_hapA.contig00792       GT------TGTTGTTGtTGtTGtTGtTGtTGtTGCTTGGATATACCTCCACTTGATCCTG
AY2_hapB.contig00598          GT------TGTTGTTGtTGtTGtTGtTGtTGtTGCTTGGATATACCTCCACTTGATCCTG
                                ^^^^^^  ^  ^
CANOR.HE681719                TCGGTGGAGGAGGCGCTCTTCTAACTGGAACAAGATCATTCTTATCACCCAGTGGTGTAG
AY2_hapA.contig00554          TCGGTGGAGGAGGCGCTCTTCTAACTGGAACAAGATCATTCTTATCACCCAGTGGTGTAG
MC0456_hapB.contig00994       TCGGTGGAGGAGGCGCTCTTCTAACTGGAACAAGATCATTCTTATCACCCAGTGGTGTAG
MC0456_hapA.contig00792       TCGGTGGAGGgGGCGCTCTTCTAACTGGAACAAGATCATTCTTgTCAtCCAGTGGTGTAG
AY2_hapB.contig00598          TCGGTGGAGGgGGCGCTCTTCTAACTGGAACAAGATCATTCTTgTCAtCCAGTGGTGTAG

CANOR.HE681719                GAACAGTGGTAGTATTTGCATTTGTTGTTCCAACACTTTTACTAAGCAAATTACTCAATG
AY2_hapA.contig00554          GAACAGTGGTAGTATTTGCATTTGTTGTTCCAACACTTTTACTAAGCAAATTACTCAATG
MC0456_hapB.contig00994       GAACAGTGGTAGTATTTGCATTTGTTGTTCCAACACTTTTACTAAGCAAATTACTCAATG
MC0456_hapA.contig00792       GAACAGTGGcgaTATTTGaATTTGTTGTTCCggCgCTTTTAtTAAGCAAATTACTCAATG
AY2_hapB.contig00598          GAACAGTGGcgaTATTTGaATTTGTTGTTCCggCgCTTTTAtTAAGCAAATTACTCAATG

CANOR.HE681719                GGGATATTGTGGTGGGGGCAGTTGCTTCGTTGCCTCCGCTTCCGTGTTGTTGAGCAGTTA
AY2_hapA.contig00554          GGGATATTGTGGTGGGGGCAGTTGCTTCGTTGCCTCCGCTTCCGTGTTGTTGAGCAGTTA
MC0456_hapB.contig00994       GGGATATTGTGGTGGGGGCAGTTGCTTCGTTGCCTCCGCTTCCGTGTTGTTGAGCAGTTA
MC0456_hapA.contig00792       GaGATATTGTGGTGGGGGCgGTTGCTTCGTTGCCTCCGCTTCCGTGTTGcTGAGCAGTTA
AY2_hapB.contig00598          GaGATATTGTGGTGGGGGCgGTTGCTTCGTTGCCTCCGCTTCCGTGTTGcTGAGCAGTTA
```

HE681723 [2 events, 1 SNP each]

```
MC0456_hapA.contig00051   AGTAGTACGGATCTACCTCATCTAAGTACAtTTCTTTAAGTTCaAAAACtCCtGAgTCGT
AY2_hapB.contig00625      AGTAGTACGGATCTACCTCATCTAAGTACAtTTCTTTAAGTTCaAAAACtCCtGAgTCGT
CANOR.HE681723            AGTAGTACGGATCTACCTCATCTAAGTACACTTCTTTAAGTTCCAAAACCCCAGAATCGT
AY2_hapA.contig00581      AGTAGTACGGATCTACCTCATCTAAGTAtACTTCTTTAAGTTCCAAAACCCCAGAATCGT
MC0456_hapB.contig00065   AGTAGTACGGATCTACCTCATCTAAGTAtACTTCTTTAAGTTCCAAAACCCCAGAATCGT
                                                        ^

MC0456_hapA.contig00051   TGTTCCCAACTGGCTcTGTATAGTTgGCCATtTTTTTCAATATCATCTCAAATCGTTTAT
AY2_hapB.contig00625      TGTTCCCAACTGGCTcTGTATAGTTgGCCATtTTTTTCAATATCATCTCAAATCGTTTAT
CANOR.HE681723            TGTTCCCAACTGGCTTTGTATAGTTAGCCATCTTTTTCAATATCATCTCAAATCGTTTAT
AY2_hapA.contig00581      TGTTCCCAACTGGCTTTGTATAGTTAGCCATCTTTTTCAATATCATCTCAAATCGTTTAT
MC0456_hapB.contig00065   TGTTCCCAACTGGCTTTGTATAGTTAGCCATCTTTTTCAATATCATCTCAAATCGTTTAT

MC0456_hapA.contig00051   CAAGAACCAGTTTTTCAGATAtGTAAGAgCATAGCTTCTTGTAACTCAAAGGTTTGAAAC
AY2_hapB.contig00625      CAAGAACCAGTTTTTCAGATAtGTAAGAgCATAGCTTCTTGTAACTCAAAGGTTTGAAAC
CANOR.HE681723            CAAGAACCAGTTTTTCAGATACGTAAGAACATAGCTTCTTGTAACTCAAAGGTTTGAAAC
AY2_hapA.contig00581      CAAGAACCAGTTTTTCAGATACGTAAGAACATAGCTTCTTGTAACTCAAAGGTTTGAAAC
MC0456_hapB.contig00065   CAAGAACCAGTTTTTCAGATACGTAAGAACATAGCTTCTTGTAACTCAAAGGTTTGAAAC

MC0456_hapA.contig00051   ACAAATGATGAATGATTTCTTTTTCGATTCTTTGTTTGGTCAATTCTGACTCTGAAAGCC
AY2_hapB.contig00625      ACAAATGATGAATGATTTCTTTTTCGATTCTTTGTTTGGTCAATTCTGACTCTGAAAGCC
CANOR.HE681723            ACAAATGATGAATGATTTCTTTTTCGATTCTTTGTTTGGTCAATTCTGACTCTGAAAGCC
AY2_hapA.contig00581      ACAAATGATGAATGATTTCTTTTTCGATTCTTTGTTTGGTCAATTCTGACTCTGAAAGCC
MC0456_hapB.contig00065   ACAAATGATGAATGATTTCTTTTTCGATTCTTTGTTTGGTCAATTCTGACTCTGAAAGCC

MC0456_hapA.contig00051   CCTTCAAGAACGAATGTTCTGTCATCATATTTATAAAtAATGTCGCACACTCtTCAAGAA
AY2_hapB.contig00625      CCTTCAAGAACGAATGTTCTGTCATCATATTTATAAAtAATGTCGCACACTCtTCAAGAA
CANOR.HE681723            CCTTCAAGAACGAATGTTCTGTCATCATATTTATAAAAAATGTCGCACACTCCTCAAGAA
AY2_hapA.contig00581      CCTTCAAGAACGAATGTTCTGTCATCATATTTATAAAAAATGTCGCACACTCCTCAAGAA
MC0456_hapB.contig00065   CCTTCAAGAACGAATGTTCTGTCATCATATTTATAAAAAATGTCGCACACTCCTCAAGAA

MC0456_hapA.contig00051   TaAAAGGTAAAGTCTGTgCATCATATGACGACTCGTTCTcTGAAGAGACCCAGTCTCCTT
AY2_hapB.contig00625      TaAAAGGTAAAGTCTGTgCATCATATGACGACTCGTTCTcTGAAGAGACCCAGTCTCCTT
CANOR.HE681723            TGAAAGGTAAAGTCTGTACATCATATGACGACTCGTTCTTTGAAGAGACCCAGTCTCCTT
AY2_hapA.contig00581      TGAAAGGTAAAGTCTGTACATCATATGACGACTCGTTCTTTGAAGAGACCCAGTCTCCTT
MC0456_hapB.contig00065   TGAAAGGTAAAGTCTGTACATCATATGACGACTCGTTCTTTGAAGAGACCCAGTCTCCTT

MC0456_hapA.contig00051   GTAGCATCCAACGgTCAAAGATCATGTATGCCACCAAATCTGGATGGCACATaCTACTgA
AY2_hapB.contig00625      GTAGCATCCAACGgTCAAAGATCATGTATGCCACCAAATCTGGATGGCACATaCTACTgA
CANOR.HE681723            GTAGCATCCAACGATCAAAGATCATGTATGCCACCAAATCTGGATGGCACATGCTACTAA
AY2_hapA.contig00581      GTAGCATCCAACGATCAAAGATCATGTATGCCACCAAATCTGGATGGCACATGCTACTAA
MC0456_hapB.contig00065   GTAGCATCCAACGATCAAAGATCATGTATGCCACCAAATCTGGATGGCACATGCTACTAA

MC0456_hapA.contig00051   ATATTTGAATCAAGAATAAATCCCTCATATACCCATACTCACGCAACCCGAGgTTTTTGT
AY2_hapB.contig00625      ATATTTGAATCAAGAATAAATCCCTCATATACCCATACTCACGCAACCCGAGgTTTTTGT
CANOR.HE681723            ATATTTGAATCAAGAATAAATCCCTCATATACCCATACTCACGCAACCCGAGTTTTTTGT
AY2_hapA.contig00581      ATATTTGAATCAAGAATAAATCCCTCATATACCCATACTCACGCAACCCGAGTTTTTTGT
MC0456_hapB.contig00065   ATATTTGAATCAAGAATAAATCCCTCATATACCCATACTCACGCAACCCGAGTTTTTTGT

MC0456_hapA.contig00051   ACATGTGCAATTGCGATTTCACAGTAAACCCATTTCGAACCCAGTAGCCACATTTTATTT
AY2_hapB.contig00625      ACATGTGCAATTGCGATTTCACAGTAAACCCATTTCGAACCCAGTAGCCACATTTTATTT
CANOR.HE681723            ACATGTGCAATTGCGATTTCACAGTAAACCCATTTCGAACCCAGTAGCCACATTTTATTT
AY2_hapA.contig00581      ACATGTGCAATTGCGATTTCACAGTAAACCCATTTCGAACCCAGTAGCCACATTTTATTT
MC0456_hapB.contig00065   ACATGTGCAATTGCGATTTCACAGTAAACCCATTTCGAACCCAGTAGCCACATTTTATTT
```

```
MC0456_hapA.contig00051     GTGACATAAGTGCTAaAGTTTTTATAGAGTAATCAAATATCAAAGACTCAACAGAaAATG
AY2_hapB.contig00625        GTGACATAAGTGCTAaAGTTTTTATAGAGTAATCAAATATCAAAGACTCAACAGAaAATG
CANOR.HE681723              GTGACATAAGTGCTAGAGTTTTTATAGAGTAATCAAATATCAAAGACTCAACAGAGAATG
AY2_hapA.contig00581        GTGACATAAGTGCTAGAGTTTTTATAGAGTAATCAAATATCAAAGACTCAACAGAGAATG
MC0456_hapB.contig00065     GTGACATAAGTGCTAGAGTTTTTATAGAGTAATCAAATATCAAAGACTCAACAGAGAATG

MC0456_hapA.contig00051     ACTCTGGCAATCCCATTCTCGTTCCCACTTTTGTAAATCTcTCAATCAAACCATCCCAGG
AY2_hapB.contig00625        ACTCTGGCAATCCCATTCTCGTTCCCACTTTTGTAAATCTcTCAATCAAACCATCCCAGG
CANOR.HE681723              ACTCTGGCAATCCCATTCTCGTTCCCACTTTTGTAAATCTATCAATCAAACCATCCCAGG
AY2_hapA.contig00581        ACTCTGGCAATCCCATTCTCGTTCCCACTTTTGTAAATCTATCAATCAAACCATCCCAGG
MC0456_hapB.contig00065     ACTCTGGCAATCCCATTCTCGTTCCCACTTTTGTAAATCTATCAATCAAACCATCCCAGG

MC0456_hapA.contig00051     CTgGAAACtGAGCATGTTCAATCAATGAGCAAATGTATGAGTGCATAGGATGAAGGAAAC
AY2_hapB.contig00625        CTgGAAACtGAGCATGTTCAATCAATGAGCAAATGTATGAGTGCATAGGATGAAGGAAAC
CANOR.HE681723              CTCGAAACCGAGCATGTTCAATCAATGAGCAAATGTATGAGTGCATAGGATGAAGGAAAC
AY2_hapA.contig00581        CTCGAAACCGAGCATGTTCAATCAATGAGCAAATGTATGAGTGCATAGGATGAAGGAAAC
MC0456_hapB.contig00065     CTCGAAACCGAGCATGTTCAATCAATGAGCAAATGTATGAGTGCATAGGATGAAGGAAAC

MC0456_hapA.contig00051     TTACATTGTCCTCATCCATGTTTGAAGCTTTCAAAACttTTCCGGTcATTGGTTCCACaA
AY2_hapB.contig00625        TTACATTGTCCTCATCCATGTTTGAAGCTTTCAAAACttTTCCGGTcATTGGTTCCACaA
CANOR.HE681723              TTACATTGTCCTCATCCATGTTTGAAGCTTTCAAAACCCTTCCGGTGATTGGTTCCACGA
AY2_hapA.contig00581        TTACATTGTCCTCATCCATGTTTGAAGCTTTCAAAACCCTTCCGGTGATTGGTTCCACGA
MC0456_hapB.contig00065     TTACATTGTCCTCATCCATGTTTGAAGCTTTCAAAACCCTTCCGGTGATTGGTTCCACGA

MC0456_hapA.contig00051     TGTTaTTTCTGTGAAcAAAACTCTTTATATCTGTATTTGCATAATCTGTCCCTTTTAGCT
AY2_hapB.contig00625        TGTTaTTTCTGTGAAcAAAACTCTTTATATCTGTATTTGCATAATCTGTCCCTTTTAGCT
CANOR.HE681723              TGTTTTTTtTGTGAAAAAAACTCTTTATATCTGTATTTGCATAATCTGTCCCTTTTAGCT
AY2_hapA.contig00581        TGTTTTTTCTGTGAAAAAAACTCTTTATATCTGTATTTGCATAATCTGTCCCTTTTAGCT
MC0456_hapB.contig00065     TGTTTTTTCTGTGAAAAAAACTCTTTATATCTGTATTTGCATAATCTGTCCCTTTTAGCT

MC0456_hapA.contig00051     CGTGAAACTCTAACGTAATCAAATATGCAATAATGTATTCAATGGCATTCAATGATTTCT
AY2_hapB.contig00625        CGTGAAACTCTAACGTAATCAAATATGCAATAATGTATTCAATGGCATTCAATGATTTCT
CANOR.HE681723              CGTGAAACTCTAACGTAATCAAATATGCAATAATGTATTCAATGGCATTCAATGATTTCT
AY2_hapA.contig00581        CGTGAAAtTCTAACGTAATCAAATATGCAATAATGTATTCAATGGCATTCAATGATTTCT
MC0456_hapB.contig00065     CGTGAAAtTCTAACGTAATCAAATATGCAATAATGTATTCAATGGCATTCAATGATTTCT
                                   ^
MC0456_hapA.contig00051     CCTgTCTGGATTGTTGGTCATTGGAcCTACTATTAAGAGTCAGGGCACTCAACTCCGCAA
AY2_hapB.contig00625        CCTgTCTGGATTGTTGGTCATTGGAcCTACTATTAAGAGTCAGGGCACTCAACTCCGCAA
CANOR.HE681723              CCTCTCTGGATTGTTGGTCATTGGATCTACTATTAAGAGTCAGGGCACTCAAtTCCGCAA
AY2_hapA.contig00581        CCTCTCTGGATTGTTGGTCATTGGATCTACTATTAAGAGTCAGGGCACTCAACTCCGCAA
MC0456_hapB.contig00065     CCTCTCTGGATTGTTGGTCATTGGATCTACTATTAAGAGTCAGGGCACTCAACTCCGCAA

MC0456_hapA.contig00051     ACTTATAAATTACCAgGACCGAATTGAAAAATGTTGAATAGTCGGGATTCTCATATTCGA
AY2_hapB.contig00625        ACTTATAAATTACCAgGACCGAATTGAAAAATGTTGAATAGTCGGGATTCTCATATTCGA
CANOR.HE681723              ACTTATAAATTACCATGACCGAATTGAAAAATGTTGAATAGTCGGGATTCTCATATTCGA
AY2_hapA.contig00581        ACTTATAAATTACCATGACCGAATTGAAAAATGTTGAATAGTCGGGATTCTCATATTCGA
MC0456_hapB.contig00065     ACTTATAAATTACCATGACCGAATTGAAAAATGTTGAATAGTCGGGATTCTCATATTCGA
```

HE681725 [3 events; 1+ SNP each]

```
MC0456_hapB.contig00381  ACATAATCGAAGTTTGATGGAATGATCTaGTTGaAATGGTAGTAATTGCTTTAGGGCATG
AY2_hapB.contig00702     ACATAATCGAAGTTTGATGGAATGATCTaGTTGaAATGGTAGTAATTGCTTTAGGGCATG
CANOR.HE681725           ACATAATCGAAGTTTGATGGAATGATCTGGTTGTAATGGTAGTAATTGCTTTAGGGCATG
AY2_hapA.contig00654     ACATAATCGAAGTTTGATGGAATGATCTGGTTGTAATGGTAGTAATTGCTTTAGGGCATG
MC0456_hapA.contig00263  ACATAATCGAAGTTTGATGGAATGATCTGGTTGTAATGGTAGTAATTGCTTTAGGGCATG

MC0456_hapB.contig00381  ATTTAAGCACAGTGCtTGATCTCAACCTTaAAGATTGAGTGGCAATTCTCTTGGCTGCAA
AY2_hapB.contig00702     ATTTAAGCACAGTGCtTGATCTCAACCTTaAAGATTGAGTGGCAATTCTCTTGGCTGCAA
CANOR.HE681725           ATTTAAGCAtAGTGCCTGATCTCAACCTTGAAGATTGAGTGGCAATTCTCTTGGCTGCAA
AY2_hapA.contig00654     ATTTAAGCACAGTGCCTGATCTCAACCTTGAAGATTGAGTGGCAATTCTCTTGGCTGCAA
MC0456_hapA.contig00263  ATTTAAGCACAGTGCCTGATCTCAACCTTGAAGATTGAGTGGCAATTCTCTTGGCTGCAA

MC0456_hapB.contig00381  CTGAACGCAATGAAtTTGGTAACaATTTtAACATTATGGTATTTAGCTTGTGATTACAGG
AY2_hapB.contig00702     CTGAACGCAATGAAtTTGGTAACaATTTtAACATTATGGTATTTAGCTTGTGATTACAGG
CANOR.HE681725           CTGAACGCAATGAACTTGGTAACGATTTCAACATTATGGTATTTAGCTTGTGATTACAGG
AY2_hapA.contig00654     CTGAACGCAATGAACTTGGTAACGATTTCAACATTATGGTATTTAGCTTGTGATTACAGG
MC0456_hapA.contig00263  CTGAACGCAATGAACTTGGTAACGATTTCAACATTATGGTATTTAGCTTGTGATTACAGG

MC0456_hapB.contig00381  TTCCTTTTAAAAGCTAAAAGCAgAGGCAAAGTAGGTCTCTTTTAAGGaTG-ATTTTTTTG
AY2_hapB.contig00702     TTCCTTTTAAAAGCTAAAAGCAgAGGCAAAGTAGGTCTCTTTTAAGGaTG-ATTTTTTTG
CANOR.HE681725           TTCCTTTTAAAAGCTAAAAGCAAAGGCAAAGTAGGTCTCTTTTAAGGGTG-ATTTTTTTG
AY2_hapA.contig00654     TTCCTTTTAAAAGCTAAAAGCAAAGGCAAAGTAGGTCTCTTTTAAGGGTGTtTTTTTTTG
MC0456_hapA.contig00263  TTCCTTTTAAAAGCTAAAAGCAAAGGCAAAGTAGGTCTCTTTTAAGGGTGTtTTTTTTTG
                                                                            ^
MC0456_hapB.contig00381  AGAAGAGG--GAGGAGTGGTAGGTCaGTGCGTGTGTaAaTGTTC-----AAAAAAAGACA
AY2_hapB.contig00702     AGAAGAGG--GAGGAGTGGTAGGTCaGTGCGTGTGTaAaTGTTC-----AAAAAAAGACA
CANOR.HE681725           AGAAGAGGGAGAGGAGTGGTAGGTCTGTGCGTGTGTGAGTGTTCaaaAAAAAAAAAGACA
AY2_hapA.contig00654     AGAAGAGGGAGAGGAGTGGTgGGTCTGTGCGTGTGTGAGTGTTC---AAAAAAAAAGACA
MC0456_hapA.contig00263  AGAAGAGGGAGAGGAGTGGTgGGTCTGTGCGTGTGTGAGTGTTC---AAAAAAAAAGACA
                                             ^
MC0456_hapB.contig00381  ATAGCTAACTACAACGCTACCACACtCATTTGAATATGT---------------------
AY2_hapB.contig00702     ATAGCTAACTACAACGCTACCACACtCATTTGAATATGT---------------------
CANOR.HE681725           ATAGCTAACcACAACGCTACCACACCCATTTGAATATGT---------------------
AY2_hapA.contig00654     ATAGCTAACTACAACGCTACCACACCCATTTGAATATGTGAATGCCCCAGATGAATATGC
MC0456_hapA.contig00263  ATAGCTAACTACAACGCTACCACACCCATTTGAATATGTGAATGCCCCAGATGAATATGC

MC0456_hapB.contig00381  GAATGCCCCAaAGGtCAATATCTGGGG-GTTAAaATCAGGCTCATTTTCgAGGTGTCTTC
AY2_hapB.contig00702     GAATGCCCCAaAGGtCAATATCTGGGG-GTTAAaATCAGGCTCATTTTCgAGGTGTCTTC
CANOR.HE681725           GAATGCCCCAGAGGACAATATCTGGGGAGTTAAGATCAGGCTCATTTTCAAGGTGTCTTC
AY2_hapA.contig00654     GAATGCCCCAGAGGACAATATCTGGGGAGTTAAGATCAGGCTCATTTTCAAGGTGTCTTC
MC0456_hapA.contig00263  GAATGCCCCAGAGGACAATATCTGGGGAGTTAAGATCAGGCTCATTTTCAAGGTGTCTTC

MC0456_hapB.contig00381  GTCCTAAAATTCGaTCAGCaTTGgCCAACATGATAAACtTCATTTTaAAGATGTAAGTCA
AY2_hapB.contig00702     GTCCTAAAATTCGaTCAGCaTTGgCCAACATGATAAACtTCATTTTaAAGATGTAAGTCA
CANOR.HE681725           GTCCTAAAATTCGGTCAGCGTTGACCAACATGATAAACCTCATTTTGAAGATGTAAGTCA
AY2_hapA.contig00654     GTCCTAAAAcTCGGTCAGCGTTGACCAACATGATAAACCTCATTTTGAAGATGTAAGTCA
MC0456_hapA.contig00263  GTCCTAAAAcTCGGTCAGCGTTGACCAACATGATAAACCTCATTTTGAAGATGTAAGTCA
                                  ^
MC0456_hapB.contig00381  ACTAAATTGTTTTGTGATATTCGCTCAAACACGCAATGCTGCAATGCATAGAGAGAACCA
AY2_hapB.contig00702     ACTAAATTGTTTTGTGATATTCGCTCAAACACGCAATGCTGCAATGCATAGAGAGAACCA
CANOR.HE681725           ACTAAATTGTTTTGTGATATTCGCTCAAACACGCAATGCTGCAATGCATAGAGAGAACCA
AY2_hapA.contig00654     ACTAAATTGTTTTGTGATATTCGCTCAAACACGCAATGCTGCAATGCATAGAGAGAACCA
MC0456_hapA.contig00263  ACTAAATTGTTTTGTGATATTCGCTCAAACACGCAATGCTGCAATGCATAGAGAGAACCA
```

```
MC0456_hapB.contig00381       TCTCGGTATATCAtCATCACAACTGGTGCCAATGTCCATTCATCTCTACAACTCAAATaG
AY2_hapB.contig00702          TCTCGGTATATCAtCATCACAACTGGTGCCAATGTCCATTCATCTCTACAACTCAAATaG
CANOR.HE681725                TCTCGGTATATCACCATCACAACTGGTGCCAATGTCCATTCATCTCTACAACTCAAATGG
AY2_hapA.contig00654          TCTCGGTATATCACCATCACAACTGGTGCCAATGTCCATTCATCTCTACAACTCAAATGG
MC0456_hapA.contig00263       TCTCGGTATATCACCATCACAACTGGTGCCAATGTCCATTCATCTCTACAACTCAAATGG

MC0456_hapB.contig00381       ATGAATTTCTGTGCATCAATTTATTCATGTTGTCAGTCaACATATCAAAGCAAAATTATT
AY2_hapB.contig00702          ATGAATTTCTGTGCATCAATTTATTCATGTTGTCAGTCaACATATCAAAGCAAAATTATT
CANOR.HE681725                ATGAATTTCTGTGCATCAATTTATTCATGTTGTCAGTCTACATATCAAAGCAAAATTATT
AY2_hapA.contig00654          ATGAATTTCTGTGCATCAATTTATTCATGTTGTCAGTCTACATATCAAAGCAAAATTATT
MC0456_hapA.contig00263       ATGAATTTCTGTGCATCAATTTATTCATGTTGTCAGTCTACATATCAAAGCAAAATTATT

MC0456_hapB.contig00381       TcAATTGAAAAATATCATCATATACAATCTCCGAGTCTCACCAGTGAAAAATCCTTcTTA
AY2_hapB.contig00702          TcAATTGAAAAATATCATCATATACAATCTCCGAGTCTCACCAGTGAAAAATCCTTcTTA
CANOR.HE681725                TTAATTGAAAAATATCATCATATACAATCTCCGAGTCTCACCAGTGAAAAATCCTTTTTA
AY2_hapA.contig00654          TTAATTGAAAAATATCATCATATACAATCTCCGAGTCTCACCAGTGAAAAATCCTTTTTA
MC0456_hapA.contig00263       TTAATTGAAAAATATCATCATATACAATCTCCGAGTCTCACCAGTGAAAAATCCTTTTTA

MC0456_hapB.contig00381       CACTTACCCTTTTAACATATGCAAGGTtCCGAACCAACTTTCTTTGTATCACTAGTAGTA
AY2_hapB.contig00702          CACTTACCCTTTTAACATATGCAAGGTtCCGAACCAACTTTCTTTGTATCACTAGTAGTA
CANOR.HE681725                CACTTACCCTTTTAACATATGCAAGGTCCCGAACCAACTTTCTTTGTATCACTAGTAGTA
AY2_hapA.contig00654          CACTTACCCTTTTAACATATGCAAGGTCCCGAACCAACTTTCTTTGTATCACTAGTAGTA
MC0456_hapA.contig00263       CACTTACCCTTTTAACATATGCAAGGTCCCGAACCAACTTTCTTTGTATCACTAGTAGTA

MC0456_hapB.contig00381       TGTTCGCTATTGAAAAACAGGATCTTGTGTCAATGCTTtCAAAATTCAAATACTAACCTA
AY2_hapB.contig00702          TGTTCGCTATTGAAAAACAGGATCTTGTGTCAATGCTTtCAAAATTCAAATACTAACCTA
CANOR.HE681725                TGTTCGCTATTGAAAAACAGGATCTTGTGTCAATGCTTCCAAAATTCAAATACTAACCTA
AY2_hapA.contig00654          TGTTCGCTATTGAAAAACAGGATCTTGTGTCAATGCTTCCAAAATTCAAATACTAACCTA
MC0456_hapA.contig00263       TGTTCGCTATTGAAAAACAGGATCTTGTGTCAATGCTTCCAAAATTCAAATACTAACCTA

MC0456_hapB.contig00381       TGTAACTAGAGCGGTGCCTGTGCTGGAACTGCCACtGATGTCGCATTCTTTCCAATtGAC
AY2_hapB.contig00702          TGTAACTAGAGCGGTGCCTGTGCTGGAACTGCCACtGATGTCGCATTCTTTCCAATtGAC
CANOR.HE681725                TGTAACTAGAGCGGTGCCTGTGCTGGAACTGCCACCGATGTCGCATTCTTTCCAATCGAC
AY2_hapA.contig00654          TGTAACTAGAGCGGcGCCTGTGCTGGAACTGCCACCGATGTCGCATTCTTTCCAATCGAC
MC0456_hapA.contig00263       TGTAACTAGAGCGGcGCCTGTGCTGGAACTGCCACCGATGTCGCATTCTTTCCAATCGAC
                                            ^
MC0456_hapB.contig00381       ACTATAAAAACaAGATTACAAGCAAAAGGTGGATTTTTcCGTAATGGAGGCTATAAAGGT
AY2_hapB.contig00702          ACTATAAAAACaAGATTACAAGCAAAAGGTGGATTTTTcCGTAATGGAGGCTATAAAGGT
CANOR.HE681725                ACTATAAAAACCAGATTACAAGCAAAAGGTGGATTTTTTCGTAATGGAGGCTATAAAGGT
AY2_hapA.contig00654          ACTATAAAAACCAGATTACAAGCAAAAGGTGGATTTTTTCGTAATGGAGGCTATAAAGGT
MC0456_hapA.contig00263       ACTATAAAAACCAGATTACAAGCAAAAGGTGGATTTTTTCGTAATGGAGGCTATAAAGGT
```

HE681722 [1 event, 1 SNP + INDEL]

```
AY2_hapA.contig00680          TGGcTTAGCCTTtaAGTTAGTTGGTaGGGGAAATAGAAGAAGAAAACGAgGTAAATTTAA
MC0456_hapA.contig00280       TGGcTTAGCCTTtaAGTTAGTTGGTaGGGGAAATAGAAGAAGAAAACGAgGTAAATTTAA
CANOR.HE681722                TGGTTTAGCCTTCCAGTTAGTTGGTGGGGGAAATAGAAGAAGAAAACGAAGTAAATTTAA
MC0456_hapB.contig00401       TGGTTTAGCCTTCCAGTTAGTTGGTGGGGGAAATAGAAGAAGAAAACGAAGTAAATTTAA
AY2_hapB.contig00728          TGGTTTAGCCTTCCAGTTAGTTGGTGGGGGAAATAGAAGAAGAAAACGAAGTAAATTTAA

AY2_hapA.contig00680          TTGGcTAGTTAATTcGTAATATAATCTTAAATAATTGGTTtTTTGTtTTATTGTGTATGG
MC0456_hapA.contig00280       TTGGcTAGTTAATTcGTAATATAATCTTAAATAATTGGTTtTTTGTtTTATTGTGTATGG
CANOR.HE681722                TTGGTTAGTTAATTGGTAATATAATCTTAAATAATcGGTTCTTTGTCTTATTGTGTATGG
MC0456_hapB.contig00401       TTGGTTAGTTAATTGGTAATATAATCTTAAATAATTGGTTCTTTGTCTTATTGTGTATGG
AY2_hapB.contig00728          TTGGTTAGTTAATTGGTAATATAATCTTAAATAATTGGTTCTTTGTCTTATTGTGTATGG

AY2_hapA.contig00680          AAAAAGTAATTAAATGATATCGTTTATAACCTA-----GAATACcCCACCGGCTGTAAAG
MC0456_hapA.contig00280       AAAAAGTAATTAAATGATATCGTTTATAACCTA-----GAATACcCCACCGGCTGTAAAG
CANOR.HE681722                AAAAAGTAATTAAATGATATCGTTTATAACCTg-----GAATACTCCACCGGCTGTAAAG
MC0456_hapB.contig00401       AAAAAGTAATTAAATGATATCGTTTATAACCTAACCTGGAATACTCgACCGGCTGTAAAG
AY2_hapB.contig00728          AAAAAGTAATTAAATGATATCGTTTATAACCTAACCTGGAATACTCgACCGGCTGTAAAG
                                                               ^^^^^        ^
AY2_hapA.contig00680          ATAAAATACAgTAATCAGAACGCAAGATCTgGATCGTTAAACAACAATACCACCtaTATG
MC0456_hapA.contig00280       ATAAAATACAgTAATCAGAACGCAAGATCTgGATCGTTAAACAACAATACCACCtaTATG
CANOR.HE681722                ATAAAATACAATAATCAGAACGCAAGATCTAGATCGTTAAACAACAATACCACCACTATG
MC0456_hapB.contig00401       ATAAAATACAATAATCAGAACGCAAGATCTAGATCGTTAAACAACAATACCACCACTATG
AY2_hapB.contig00728          ATAAAATACAATAATCAGAACGCAAGATCTAGATCGTTAAACAACAATACCACCACTATG
```

HE681719 [1 event, 2 SNPs]

```
AY2_hapA.contig00617          CGTGTGACTTtAAAATTGAGaCAAAAtTTGTGaCAAATTGgTGTAGTgcGAAGAGGaGTA
MC0456_hapB.contig00752       CGTGTGACTTtAAAATTGAGaCAAAAtTTGTGaCAAATTGgTGTAGTgcGAAGAGGaGTA
CANOR.HE681719                CGTGTGACTTC-AAATTGAGTCAAAACTTGTGGCAAATTGATGTAGTCTGAAGAGGCGTA
MC0456_hapA.contig00572       CGTGTGACTTC-AAATTGAGTCAAAACTTGTGGCAAATTGATGTAGTCTGAAGAGGCGTA
AY2_hapB.contig00662          CGTGTGACTTC-AAATTGAGTCAAAACTTGTGGCAAATTGATGTAGTCTGAAGAGGCGTA

AY2_hapA.contig00617          AGTcCTAGCTCGATTATACCTCTATAATaTATTGcAaTGGTTCGTTGGTTGGCTGGTTAA
MC0456_hapB.contig00752       AGTcCTAGCTCGATTATACCTCTATAATaTATTGcAaTGGTTCGTTGGTTGGCTGGTTAA
CANOR.HE681719                AGTTCTAGCTCGATTATACCTCTATAATCTATTGTAGTGGTTCGTTGGTTGGCTGGTTAA
MC0456_hapA.contig00572       AGTTCTAGCTCGATTATACCTCTATAATCTAcaGTAGTGGTTCGTTGGTTGGCTGGTTAA
AY2_hapB.contig00662          AGTTCTAGCTCGATTATACCTCTATAATCTAcaGTAGTGGTTCGTTGGTTGGCTGGTTAA
                                                             ^^
AY2_hapA.contig00617          TGCCCTTGGCTCTcttAATtCTCTtAAAGTTTTTGCAACACAATTATTTGTTGCAACAAA
MC0456_hapB.contig00752       TGCCCTTGGCTCTcttAATtCTCTtAAAGTTTTTGCAACACAATTATTTGTTGCAACAAA
CANOR.HE681719                TGCCCTTGGCTCTTCCAATCCTCTGAAAGTTTTTGCAACACAATTATTTGTTGCAACAAA
MC0456_hapA.contig00572       TGCCCTTGGCTCTTCCAATCCTCTGAAAGTTTTTGCAACACAATTATTTGTTGCAACAAA
AY2_hapB.contig00662          TGCCCTTGGCTCTTCCAATCCTCTGAAAGTTTTTGCAACACAATTATTTGTTGCAACAAA
```

HE681721 [5 events, 7 SNPs]

```
AY2_hapA.contig00988        CAAaTAAATtTCAAAtAAATTTAATAGTCTtGtTATAATTGACCAGTCTTtAAATAACTa
MC0456_hapA.contig00568     CAAaTAAATtTCAAAtAAATTTAATAGTCTtGtTATAATTGACCAGTCTTtAAATAACTa
CANOR.HE681721              CAAGTAAATCTCAAACAAATTTAATAGTCTAGCTATAATTGACCAGTCTTCAAATAACTC
MC0456_hapB.contig00748     CAAGTAAATCTCAAACAAATTTAATAGTCTAGCTATAATTGACCAGTCTTCAAATAACTC
AY2_hapB.contig01037        CAAGTAAATCTCAAACAAATTTAATAGTCTAGCTATAATTGACCAGTCTTCAAATAACTC

AY2_hapA.contig00988        TCAGTGGAgCAAACTAAAGCACTCtACCTCGGAAATGCAGACATACAGGCTCATTCCCTG
MC0456_hapA.contig00568     TCAGTGGAgCAAACTAAAGCACTCtACCTCGGAAATGCAGACATACAGGCTCATTCCCTG
CANOR.HE681721              TCAtTGGAACAAACTAAAGcGCTCCACCTCGGAAATGCAGACATACAGGCT--TTCCCTG
MC0456_hapB.contig00748     TCAGTGGAACAAACTAAAcCACTCCACCTCGGAAATGCAGACATACAGGCTCATTCCCTG
AY2_hapB.contig01037        TCAGTGGAACAAACTAAAcCACTCCACCTCGGAAATGCAGACATACAGGCTCATTCCCTG
                                              ^

AY2_hapA.contig00988        TTGGaATTTTGGTTACGTTATCTTTGCTTGAcGAAAGTAtTTCCTCTGCAACTTGTAGCA
MC0456_hapA.contig00568     TTGGaATTTTGGTTACGTTATCTTTGCTTGAcGAAAGTAtTTCCTCTGCAACTTGTAGCA
CANOR.HE681721              TTGGGATTTTGGTTAtGTTATCTTTGCTTGATGAAAGTACTTCCTCTGCAACTTGTAGCA
MC0456_hapB.contig00748     TTGGGATTTTGGTTACGTTATCTTTGCTTGATGAAAGTACTTCtTCT-CAACTTGTAGCA
AY2_hapB.contig01037        TTGGGATTTTGGTTACGTTATCTTTGCTTGATGAAAGTACTTCtTCT-CAACTTGTAGCA
                                                                       ^

AY2_hapA.contig00988        GCTTGCACACTTgAtTAACTAGTcTAACAGGATCAAAGCAACACATTTATAGCAgCtcCA
MC0456_hapA.contig00568     GCTTGCACACTTgAtTAACTAGTcTAACAGGATCAAAGCAACACATTTATAGCAgCtcCA
CANOR.HE681721              GCTTGCACACTTAACTAACTAGTGTAACAGGATCAAAGCgACACATTTATAGCAACAGCA
MC0456_hapB.contig00748     GCTTGCACgCTTAACTAACTAGTGTAACAGGATCAAAGCAACACATTTATAGCAACAGCA
AY2_hapB.contig01037        GCTTGCACgCTTAACTAACTAGTGTAACAGGATCAAAGCAACACATTTATAGCAACAGCA
                                    ^

AY2_hapA.contig00988        TGaCCgAATACtTATCCATATaATGtTTCTAACAATAGTTTGCAGTAAGAGTGACTCGTA
MC0456_hapA.contig00568     TGaCCgAATACtTATCCATATaATGtTTCTAACAATAGTTTGCAGTAAGAGTGACTCGTA
CANOR.HE681721              TGGCCAAATACATATtCATATGATGCcTCTAACAATAGTTTGCAGTAAGAGTGACTCGTA
MC0456_hapB.contig00748     TGGCaAAATACATATCCATATGATGCTTCTAACAAcAGTTTGCAGTAAGAGTGACTCGTA
AY2_hapB.contig01037        TGGCaAAATACATATCCATATGATGCTTCTAACAAcAGTTTGCAGTAAGAGTGACTCGTA
                                                               ^

AY2_hapA.contig00988        CCCTTTTCATTTGGTGTTGCAAAATGTTAGTCGTGTCCAGTGTGACGTATTGCCCCATCA
MC0456_hapA.contig00568     CCCTTTTCATTTGGTGTTGCAAAATGTTAGTCGTGTCCAGTGTGACGTATTGCCCCATCA
CANOR.HE681721              CCCTTTTCATTTGGTGTTGCAAAATGTTAGTCGTGTCCAGTGTGACGTATTGCCCCATCg
MC0456_hapB.contig00748     CtCTTTTCATTTGGTGTTGCAAAATGTTAGTCGTGTCCAGTGTGACGTATTGCCCCATCA
AY2_hapB.contig01037        CtCTTTTCATTTGGTGTTGCAAAATGTTAGTCGTGTCCAGTGTGACGTATTGCCCCATCA
                             ^

AY2_hapA.contig00988        AAAAAAGAAGCCTTAGTCCCGCAAAaCGTTCACCCGCCAAACGACCCAACTAAAATAATA
MC0456_hapA.contig00568     AAAAAAGAAGCCTTAGTCCCGCAAAaCGTTCACCCGCCAAACGACCCAACTAAAATAATA
CANOR.HE681721              AAAAAAGAAGCCTTAGTCCCGCAAAGCGTTCACCCGCCAAACGACCCAACTAAAATAATA
MC0456_hapB.contig00748     AAAAAAGAAGCCTTAGTCCCGCAAAGCGTTCACCCGCCAAACGACCCAACTAAAATAATA
AY2_hapB.contig01037        AAAAAAGAAGCCTTAGTCCCGCAAAGCGTTCACCCGCCAAACGACCCAACTAAAATAATA

AY2_hapA.contig00988        ATCACCCAATTTTTAGCTGAGTTTATTGCATAGAAGGGTACATTGTCACAAGCACCtTAA
MC0456_hapA.contig00568     ATCACCCAATTTTTAGCTGAGTTTATTGCATAGAAGGGTACATTGTCACAAGCACCtTAA
CANOR.HE681721              ATCACCCAATTTTTAGCTGAGTTTATTGCATAGAAGGGTACATTGTCACAAGCACCCTAA
MC0456_hapB.contig00748     ATtACCCAATTTTTAGCTGAGTTTATTGCATAGAAGGGTACATTGTCACAAGCACCCTAA
AY2_hapB.contig01037        ATtACCCAATTTTTAGCTGAGTTTATTGCATAGAAGGGTACATTGTCACAAGCACCCTAA
                              ^

AY2_hapA.contig00988        AAtaCaCGGAACCACtAACACCAATGTCACCACCAGGTCAAAAA----------------
MC0456_hapA.contig00568     AAtaCaCGGAACCACtAACACCAATGTCACCACCAGGTCAAAAAAACTTCCATACTGCCA
CANOR.HE681721              AgCCCTCGGAACCACCAACACCAATGTCACCACCAGGTCAAAAAAACTTCCATACTGCCA
MC0456_hapB.contig00748     AACCCTCGGAAtCACCAACACCAATGTCACCACCAGGTCAAAAAAACTTCCATACTGCCA
AY2_hapB.contig01037        AACCCTCGGAAtCACCAACACCAATGTCACCACCAGGTCAAAAA----------------
                                       ^
```

HE681721 [1 event, 1 SNP + LOH

```
AY2_hapA.contig00475      TGGAAACtAAAACACTATTTcGaATtTGACCATAACCTCCTTCCAACCCATCACCTTCTT
MC0456_hapB.contig00987   TGGAAACtAAAACACTATTTcGaATtTGACCATAACCTCCTTCCAACCCATCACCTTCTT
CANOR.HE681721            TGGAAACCAAAACACTATTTTGGATATGACCATAACCTCCTTCCAACCCATCACCTTCTT
MC0456_hapA.contig00785   TGGAAACCAAAACACTATTTTGGATATGACCATAACCTCCTTCCAACCCATCACCTTCTT
AY2_hapB.contig00517      TGGAAACCAAAACACTATTTTGGATATGACCATAACCTCCTTCCAACCCATCACCTTCTT

AY2_hapA.contig00475      CGGGGGCGTAAATTCTATATATACGCAAGAATACaCCACATTGTAACATTGGAATACTAA
MC0456_hapB.contig00987   CGGGGGCGTAAATTCTATATATACGCAAGAATACaCCACATTGTAACATTGGAATACTAA
CANOR.HE681721            CGGGGGCGTAAATTCTATATATACGCAAGAATACCCCACATTGTAACATTGGAATACTAA
MC0456_hapA.contig00785   CGGGGGCGTAAATTCTATATATACGCAAGAATACCCCACATTGTAACATTGGAATACcAA
AY2_hapB.contig00517      CGGGGGCGTAAATTCTATATATACGCAAGAATACCCCACATTGTAACATTGGAATACcAA
                                                                                   ^
AY2_hapA.contig00475      CTCtTCCCAATCTCTCTAAATGACGAAAACAAGCTTGGGAAATTTCTGCAATGCCAATTT
MC0456_hapB.contig00987   CTCtTCCCAATCTCTCTAAATGACGAAAACAAGCTTGGGAAATTTCTGCAATGCCAATTT
CANOR.HE681721            CTCGTCCCAATCTCTCTAAATGACGAAAACAAGCTTGGGAAATTTCTGCAATGCCAATTT
MC0456_hapA.contig00785   CTCGTCCCAATCTCTCTAAATGACGAAAACAAGCTTGGGAAATTTCTGCAATGCCAATTT
AY2_hapB.contig00517      CTCGTCCCAATCTCTCTAAATGACGAAAACAAGCTTGGGAAATTTCTGCAATGCCAATTT

AY2_hapA.contig00475      CGATTTGATTTAACAAGAGAAAtTTCTTTTCCGCTTCATCAGAAGTGAGGTTAGTTTTGG
MC0456_hapB.contig00987   CGATTTGATTTAACAAGAGAAACTTCTTTTCCGCTTCATCAGAAGTGAGGTTAGTTTTGG
CANOR.HE681721            CGATTTGATTTAACAAGAGAAAtTTCTTTTCCGCTTCATCAGAAGTGAGGTTAGTTTTGG
MC0456_hapA.contig00785   CGATTTGATTTAACAAGAGAAACTTCTTTTCCGCTTCATCAGAAGTGAGGTTAGTTTTGG
AY2_hapB.contig00517      CGATTTGATTTAACAAGAGAAACTTCTTTTCCGCTTCATCAGAAGTGAGGTTAGTTTTGG
                                                ^
AY2_hapA.contig00475      CGAGAATTTTTTCATTGTGGAATCCTCGATTATGAAATAAGGACAAGTAGGATAaTCTAA
MC0456_hapB.contig00987   CGAGAATTTTTTCATTGTGGAATCCTCGATTATGAAATAAGGACAAGTAGGATAaTCTAA
CANOR.HE681721            CGAGAATTTTTTCATTGTGGAATCCTCGATTATGAAATAAGGACAAGTAGGATAGTCTAA
MC0456_hapA.contig00785   CGAGAATTTTTTCATTGTGGAATCCTCGATTATGAAATAAGGACAAGTAGGATAGTCTAA
AY2_hapB.contig00517      CGAGAATTTTTTCATTGTGGAATCCTCGATTATGAAATAAGGACAAGTAGGATAGTCTAA

AY2_hapA.contig00475      GCATTATAAACAAgATTCCAATTTGAGCAAAGTCACCaCGATGTGCaACTTTAATATCTG
MC0456_hapB.contig00987   GCATTATAAACAAgATTCCAATTTGAGCAAAGTCACCaCGATGTGCaACTTTAATATCTG
CANOR.HE681721            GCATTATAAACAAAATTCCAATTTGAGCAAAGTCACCGCGATGTGCGACTTTAATATCTG
MC0456_hapA.contig00785   GCATTATAAACAAAATTCCAATTTGAGCAAAGTCACCGCGATGTGCGACTTTAATATCTG
AY2_hapB.contig00517      GCATTATAAACAAAATTCCAATTTGAGCAAAGTCACCGCGATGTGCGACTTTAATATCTG
```
